# Genome and Pangenome Analysis of *Lactobacillus hilgardii* FLUB—A New Strain Isolated from Mead

**DOI:** 10.3390/ijms22073780

**Published:** 2021-04-06

**Authors:** Klaudia Gustaw, Piotr Koper, Magdalena Polak-Berecka, Kamila Rachwał, Katarzyna Skrzypczak, Adam Waśko

**Affiliations:** 1 Department of Biotechnology, Microbiology and Human Nutrition, Faculty of Food Science and Biotechnology, University of Life Sciences in Lublin, Skromna 8, 20-704 Lublin, Poland; magdalena.polak-berecka@up.lublin.pl (M.P.-B.); kamila.rachwal@up.lublin.pl (K.R.); adam.wasko@up.lublin.pl (A.W.); 2 Department of Genetics and Microbiology, Institute of Biological Sciences, Maria Curie-Skłodowska University, Akademicka 19, 20-033 Lublin, Poland; 3 Department of Fruits, Vegetables and Mushrooms Technology, Faculty of Food Science and Biotechnology, University of Life Sciences in Lublin, Skromna 8, 20-704 Lublin, Poland; katarzyna.skrzypczak@up.lublin.pl

**Keywords:** whole genome sequencing, pangenome, spoilage, fructophilicity, mead, honey, traditional fermentation

## Abstract

The production of mead holds great value for the Polish liquor industry, which is why the bacterium that spoils mead has become an object of concern and scientific interest. This article describes, for the first time, *Lactobacillus hilgardii* FLUB newly isolated from mead, as a mead spoilage bacteria. Whole genome sequencing of *L. hilgardii* FLUB revealed a 3 Mbp chromosome and five plasmids, which is the largest reported genome of this species. An extensive phylogenetic analysis and digital DNA-DNA hybridization confirmed the membership of the strain in the *L. hilgardii* species. The genome of *L. hilgardii* FLUB encodes 3043 genes, 2871 of which are protein coding sequences, 79 code for RNA, and 93 are pseudogenes. *L. hilgardii* FLUB possesses three clustered regularly interspaced short palindromic repeats (CRISPR), eight genomic islands (44,155 bp to 6345 bp), and three (two intact and one incomplete) prophage regions. For the first time, the characteristics of the genome of this species were described and a pangenomic analysis was performed. The concept of the pangenome was used not only to establish the genetic repertoire of this species, but primarily to highlight the unique characteristics of *L. hilgardii* FLUB. The core of the genome of *L. hilgardii* is centered around genes related to the storage and processing of genetic information, as well as to carbohydrate and amino acid metabolism. Strains with such a genetic constitution can effectively adapt to environmental changes. *L. hilgardii* FLUB is distinguished by an extensive cluster of metabolic genes, arsenic detoxification genes, and unique surface layer proteins. Variants of MRS broth with ethanol (10–20%), glucose (2–25%), and fructose (2–24%) were prepared to test the strain’s growth preferences using Bioscreen C and the PYTHON script. *L. hilgardii* FLUB was found to be more resistant than a reference strain to high concentrations of alcohol (18%) and sugars (25%). It exhibited greater preference for fructose than glucose, which suggests it has a fructophilic nature. Comparative genomic analysis supported by experimental research imitating the conditions of alcoholic beverages confirmed the niche specialization of *L. hilgardii* FLUB to the mead environment.

## 1. Introduction

Mead is considered to be one of the oldest fermented products, with written sources dating back to the Roman period [1]. In Poland, mead is not only a standard food product: the consumption of this beverage has a cultural and a historical aspect, similar to pulque in Mexico [2]. Mead is divided into grades depending on the volume ratio of honey to water. The noblest meads are those least diluted with water, at a ratio of one part honey to half a part water; other proportions are 1:1, 1:2, and 1:3 [3]. The honey-to-water ratio determines the alcohol content of a mead, which usually ranges from 8 to 18% [4]. Meads which are produced by traditional methods are registered as “Traditional Speciality Guaranteed” products by the European Union. Since 2017, they have also been registered as a “Traditional Old Polish” brand (półtorak/dwójniak/trójniak/czwórniak staropolski tradycyjny, depending on their grade) [5]. The composition of mead is mainly ethanol, a mixture of carbohydrates, chiefly glucose and fructose, and other substances such as organic acids, amino acids, minerals, phenolic acid, and flavonoids [3,6]. The environment of mead, with its high concentrations of sugars and the presence of ethanol, is unfavorable for the development of undesirable microorganisms [7], especially considering the fact that honey is recognized as an antimicrobial substance [8,9,10]. Because mead has a long tradition of production, an inviolable recipe, and antimicrobial properties, the source of spoiled mead has become an object of interest. In this study, attention was paid to a new strain of *L. hilgardii* FLUB, which was found to be the cause of spoilage of meads taken from the production line in one of the factories in Poland. Although ubiquitous lactic acid bacteria (LAB) are commonly considered to be beneficial microbes, some of them may cause undesirable changes in flavor and texture as they metabolize and multiply in food commodities. The species *L. hilgardii* (presently *Lentilactobacillus hilgardii*), which is the subject of this paper, was originally isolated by Couto and Hogg [11] from port wine, in which it was the main source of spoilage compounds [12]. This strain is known for its ability to survive and multiply in wines during maturation. The bacteria are resistant to relatively high concentrations of ethanol, acidic environments, and sulfates [13], and therefore present a new risk to the mead industry. The mead environment creates unfavorable conditions for the multiplication of bacteria. These conditions have shaped the mechanisms which allow *L. hilgardii* FLUB to colonize this niche and distinguish it significantly from *L. hilgardii* NRRL B-1843 (DSMZ 20176, ATCC 8290, JCM 1155) isolated from wine, which is used as the reference strain in this article.

This paper presents a comprehensive overview of the whole genome of *L. hilgardii* FLUB, provides an insight into its phylogenetics, and reports experiments reproducing the environmental conditions of its growth. A comparative genome and pangenome analysis was performed to demonstrate the variability (and novelty) of the FLUB strain related to its adaptation to the mead environment. We used the concept of the pangenome not only to establish the common features of all publicly available genomes of *L. hilgardii* species, but also, on the basis of differences between the strains, to describe the unique traits of *L. hilgardii* FLUB which allow it to occupy the new niche provided by mead.

## 2. Results and Discussion

### 2.1. Isolation and Identification

Samples of microorganisms from the main stages of production of mead were purified and assigned to ten species using the MALDI-Biotyper. The isolates were identified by MALDI-TOF, and the spectra obtained were aligned with the Bruker database. The spectra of the isolated strains had a high probability of identification of over two points (the experiments were carried out in triplicate). The analysis of the spectra showed that they corresponded to one *L. hilgardii* strain, and 25 other strains belonging to the species *Acetobacter pasteurianus, Staphylococcus epidermidis, S. capitis, S. hominis ssp. novobiosepticus, Stenotrophomonas maltophilia, Burkholderia phymatum, Bacillus simplex, Micrococcus luteus*, and *Bacteroides massiliensis*. All the species, except for *A. pasteurianus* and *L. hilgardii*, were considered as contamination originating from air or human skin. The strains were tested for growth at high sugar or alcohol concentrations. The isolates regarded as contamination were able to survive in these conditions, but could not multiply. Therefore, it was concluded that *A. pasteurianus* and *L. hilgardii* were responsible for the spoilage of the mead. Since *L. hilgardii* is a lactic acid bacterium which usually occurs in wines, this isolate was selected for further analysis. The presence of *L. hilgardii* in mead can pose a serious problem for this branch of the alcohol industry. Although the occurrence of spoilage strains in mead is a novelty, the sugar-rich mead environment bears similarity to the environment of port wine, which is likewise considered to be rich in sugars [14] and has been reported to be spoilt by *L. hilgardii*. Some bacteria have also been isolated from honey, which is generally regarded as a microbiologically stable substance; they included *Bacillus cereus, B. pumilus, B. coagulans, Escherichia coli*, and some species of *Enterobacter, Clostridium, Proteus*, and *Klebsiella* [15].

**Figure 1:**
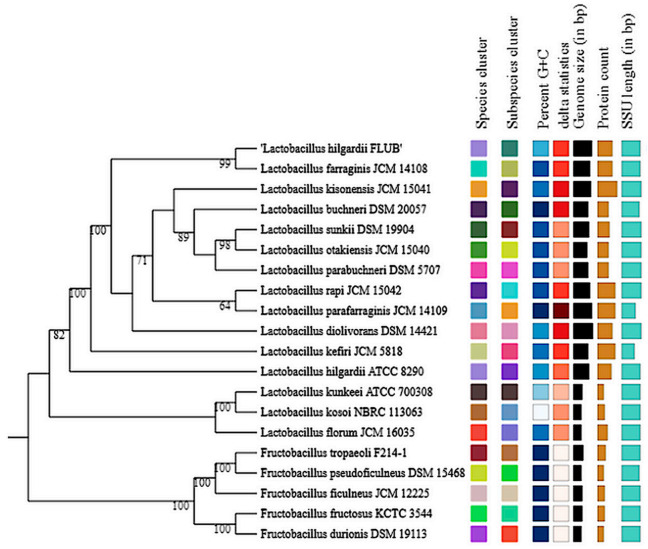
Tree inferred with FastME 2.1.6.1 from GBDP distances calculated from 16S rDNA gene sequences. The branch lengths are scaled in terms of the GBDP distance formula d5. The numbers above the branches are GBDP pseudo-bootstrap support values >60% from 100 replications, with an average branch support of 83.1%. The tree was rooted at the midpoint.

Considering that the isolate FLUB has multiple strain-specific features, a comprehensive phylogenetic analysis was performed using the conventional phylogenetic approach based on the 16S rRNA gene. It confirmed the assignment of the strain FLUB to the *L. hilgardii* species. The analysis of the 16S rRNA gene sequence alone also showed that *L. hilgardii* FLUB was phylogenetically the closest to *L. farraginis* JCM 14108, followed by *L. kisonensis* JCM 15,041 and *L. buchneri* DSM 20,057 (Figure 1). A δ value of 0.35 indicated the potential for errors in treelikeness [16], with a relatively low average branch support (54.7%).

**Figure 2:**
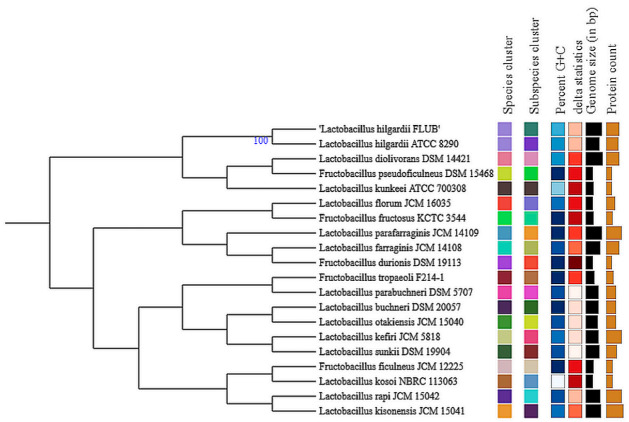
Tree inferred with FastME 2.1.6.1 from GBDP distances calculated from genome sequences. The branch lengths are scaled in terms of GBDP distance formula d5. The numbers above the branches are GBDP pseudo-bootstrap support values >60% from 100 replications, with an average branch support of 16.6%. The tree was rooted at the midpoint.

When whole genomes were compared, the newly isolated strain FLUB appeared most similar to *L. hilgardii* ATCC 8290, and then to *L. diolivorans* DSM 14,421 (Figure 2). BLAST pairwise comparisons aligned the strain FLUB with *L. hilgardii* ATCC 8290 (dDDH d0 66.8%, d4 76.5%, and a G+C difference of 0.48%) and with *L. paraplantarum* (dDDH d0 13%, d4 35.3%, and a G+C difference of 3.6%). In terms of the source of isolation, there was a clear similarity with *L. farraginis*, which had been isolated from residues from the production of shochu, a traditional Japanese alcoholic beverage [17].

**Figure 3:**
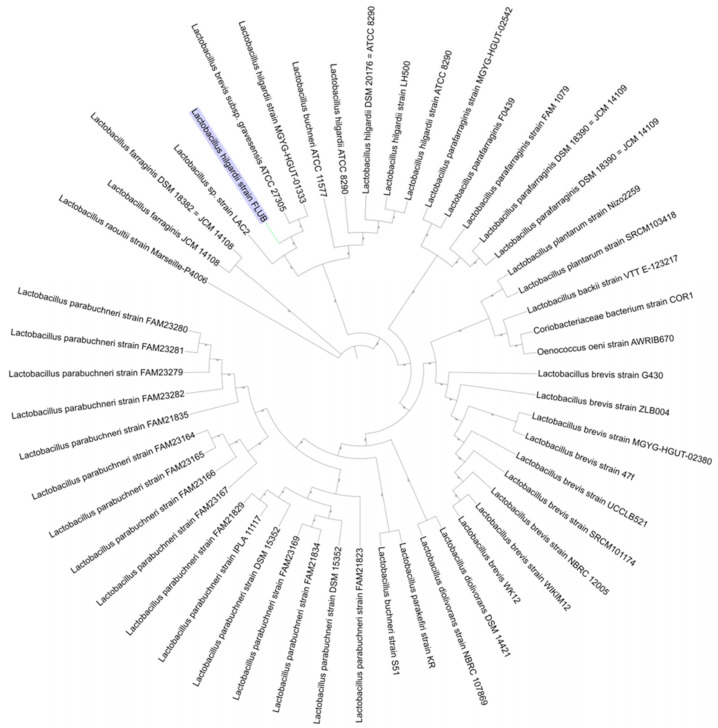
Phylogenetic tree based on PGFams, visualized in iTOL.

Subsequently, a phylogenetic tree was generated based on the PATRIC Codon Tree workflow (Supplementary Materials), where the RAxML program compares and analyzes a single copy of PGFams to coding DNA and proteins. The comparison at the level of protein families and corresponding sequences introduced a phylogenetic consensus. The strains closest related to the strain FLUB were *L. hilgardii* MGYG-HGUT-01333 and *L. brevis subsp. gravesensis* ATCC 27,305 (also known in NCBI GenBank as *L. hilgardii* LMG 07934 NZ_CP050262; this latter name was used in the pangenome study). Trees exported in Newark format and visualized in iTOL are shown in Figure 3 [18].

Although average nucleotide identity (ANI) does not implement paralogous gene filtering for each predicted dDDH value, this analysis has located much closer relatives. The service measures ANI based on BLAST+(ANIb) and MUMmer (ANIm) as well as correlation indexes of tetra-nucleotide signatures [19]. At 99.909% nucleotide identity, *L. brevis* subsp. *gravesensis* ATCC 27,305 was again the closest phylogenetic neighbor of the investigated strain. A similar, MinHash analysis performed using PATRIC’s Similar Genome Finder [20,21] indicated that *L. hilgardii* FLUB shared the same degree of phylogenetic affinity (912/1000) with *L. hilgardii* MGYG-HGUT-01333 and *L. brevis* subsp. *gravesensis* ATCC 27305. Interestingly, these two last strains had both been isolated from human intestines. The next in line were *lactobacilli* isolated from fermentation residues (strain LAC2), while the isolates of *L. hilgardii* species from wine had a very low similarity score (383/1000). This illustrates exactly how much *L. hilgardii* FLUB originating from mead differs from other representatives of its species.

### 2.2. General Genome Features

**Figure 4:**
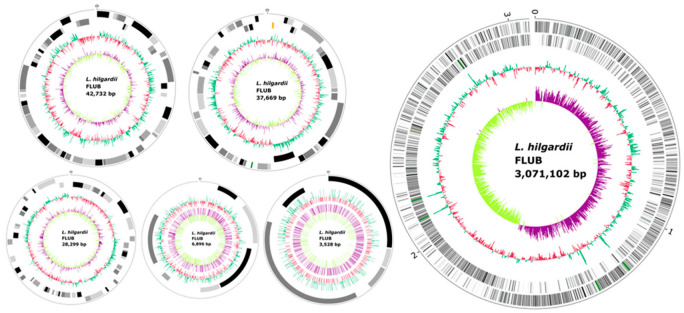
Circular map of the *L. hilgardii* FLUB chromosome and plasmids. The outer circle shows the scale in megabases (Mb). The representation, from outer to inner circle, is as follows: forward and reverse strand CDSs (the color gradient represents the percentage of GC; the green stripes represent RNAs genes), GC content, and GC skew. The genome map was visualized using the CGView circular genome visualization tool.

The whole genome sequence of *L. hilgardii* FLUB is a circular chromosomal sequence of 3,071,102 nucleotides and 5 plasmids; in total, it comprises 3,190,226 nucleotides. A circular graphical display of the distribution of genome annotations generated by CGView is shown in Figure 4.

The genome of *L. hilgardii* can be classified as a large genome when compared to other LAB genomes [22]. The longest sequence in the chromosome has 3,071,102 base pairs, and the longest sequence among plasmids, located on plasmid 1, is 42,732 bp. Moderate N50 values (3 Mbp for the chromosome, and a range of 42–43 kbp for the plasmids) may indicate that very short contigs were correctly removed from the genome assembly. The GC content of the complete genome is on average 40.1 mol%. In total, 84.1% of the genes carry information about protein coding, with a chromosome ratio of 84.7%. The gap ratio (0%) in the sequence, both on the chromosome and the plasmids, confirms the good selection of the sequencing techniques. Hybrid long-read sequencing by MinION together with Illumina short-read are increasingly being used to avoid incomplete constructions that hinder complete genome assembly [23,24,25]. The GC content in the sequences ranges from 35.6 to 41.5%, and is 40.1% for the whole genome. Based on a comparison of GC content between strains, it can be determined whether a particular strain belongs to a species [26,27]. The standard deviation for all the *L. hilgardii* genomes for this parameter is 0.2%, which confirms the assumption that all the investigated strains belong to the same species. The genome of *L. hilgardii* FLUB has 2997 protein coding sequences (CDS) (3136 PATRIC CDS and 0 partial CDS), 61 transfer RNA (tRNA) genes, and 15 ribosomal RNA (rRNA) genes (0 Miscellaneous RNA). The genome contains a set of tRNA genes for 20 basic amino acids, Lys (tree repetitions tRNA-encoding genes), Ile (4), Ala (2), Val (3), Thr (4), Gly (4), Leu (5), Arg (6), Pro (3), Asp (3), Phe (2), Glu (3), Asn (4), Tyr (2), Trp, His, Gln (3), and Cys. Makarova et al. described the relationship between the number of mRNA and tRNA genes and the better adaptation of the strain to the environment. The FLUB strain, compared to the other strains of *hilgardii* has a relatively high number of these genes [22,28]. The features annotated by Dfast are summarized in Table 1.

The annotation includes 1018 hypothetical proteins and 2118 proteins with functional assignments. The proteins with functional assignments are 729 proteins with enzyme commission (EC) numbers [29], 636 proteins with gene ontology (GO) assignments [30], and 545 proteins mapped to KEGG pathways [31]. The PATRIC annotation for the genome of *L. hilgardii* FLUB includes two types of protein families [32]: 2901 proteins that belong to genus-specific protein families (PLFams) and 2929 proteins that belong to cross-genus protein families (PGFams). Certain fragments of the genes in the bacterial genome are not functional; FLUB harbors 93 pseudogenes. The number of pseudogenes in LAB ranges from 20 to over 200 [22]. Ochman and Davalos [33] have noted that there is no space for large amounts of pseudogenes in the genomes of free-living bacteria, and so they are removed by effective selection. Three regularly interspaced short palindromic repeats (CRISPR) were found in the whole genome sequence (WGS): one located on the circular chromosomal sequence CDS (3,071,102 bp, locus 2,452,162–2,459,226, with a prediction of genes of csn2, cas2, cas1, cas9), and two on plasmids 2 (37,669 bp, locus 425–582) and 4 (6896 bp, locus 5201–5303), indicating that *L. hilgardii* FLUB may acquire resistance to phage infection [34,35]. In this article, we wanted to identify the characteristics of *L. hilgardii* FLUB that may be associated with its evolutionary success in adapting to the new habitat provided by mead. To this end, we scanned the FLUB genome for the presence of genome islands (GIs), which are considered to be fragments/clusters probably acquired by horizontal transfer. They increase the adaptability of the bacteria to the environment, include new genes, metal resistance genes, additional metabolic pathways, and antimicrobial resistance genes. An IslandViewer 4 analysis was performed for the FLUB genome using a combination of IslandPath-DIMOB, IslandPick, and SIGI-HMM prediction methods. Based on the presence of mobility genes and nucleotide bias (IslandPath-DIMOB), only eight GIs were detected, ranging in length from 6345 bp to 44,155 bp [36]. Within these genomic islands, there are likely to be acquired genes, which is an important factor in the “novelty” of this strain. The genomic islands include, among others, nine transporter genes, a cobalt ATP-binding cassette (ABC) transporter, a major facilitator superfamily (MFS) transporter, and three ECF transporter genes involved in the vitamin and micronutrient uptake in bacteria. This is particularly interesting, and essential to the FLUB strain, as nutrient uptake in dilute alcoholic beverage environments is impaired. Two genes encode mscL, of large-conductance mechanosensitive channel, a membrane protein responsible for the cell’s response to osmotic stress, which remarkably suited the environment in which the FLUB strain lives [37]. Genes encoding three glycosyltransferases are also present; with their contribution it is possible to break down the glycosidic bonds of various oligosaccharides, compounds found in nature and plants. Along with the mannosyl-glycoprotein endo-beta-N-acetylglucosamidase gene, they enrich the ability of the FLUB strain to degrade sugars [38]. As easily predicted, phage genes are present within the genomic islands, with eight genes related to capsid, tail proteins, or tape measure proteins, as genetic material due to horizontal transfer constantly mixes with the host genome. Additionally, among the genes located within the genomic islands, 93 genes (37% of the total), are unidentified genes of unknown function, where some unexplored potential of this strain resides.

### 2.3. Plasmids

The *L. hilgardii* FLUB has five plasmids with sizes of 42,732 bp, 37,669 bp, 28,299 bp, 6896 bp, and 3528 bp (interactive Krona graphs for each plasmid are available in the Supplementary Materials, and more). Plasmids ranging in size from 3 to 200 kb are common in LAB. The proportion of genes encoded by plasmids in LAB ranges from 0–4.8% of the total gene content [22]. In the tested strain, 2.55% of the genes are encoded on plasmids. Multiple sequence alignment was performed for replication proteins derived from individual plasmids (figure in the supplement). It clearly shows that the replicases of the four largest plasmids are very similar to each other, although they represent different groups of incompatibility. They were all classified into the same protein family under the FIGFams classification. In the case of the smallest plasmid the replicase sequence is significantly different from the others, which suggests a different origin of this plasmid and probably a relatively recent evolutionary appearance in the genome. It appears that all plasmids replicate themselves in the presence of rep proteins, together with several host proteins encoded on the chromosome (DnaA, DnaB, primase, polymerase DNA). All plasmids have a gene identified as a plasmid replication initiation protein (plasmid 1—GQR93_14535 and also two helix-turn-helix domains; plasmid 2—GQR93_14790 with a helix-turn-helix domain responsible for iteron interaction; plasmid 3—GQR93_15020; plasmid 4—GQR93_15175; and plasmid 5—GQR93_15195). Plasmids of this species have been described previously. Lucas et al. described plasmid pHDC from *L. hilgardii* IOEB 0006 encoding the histidine decarboxylation system [39], and Josson et al. described pLAB1000 [40]. These two plasmids bear no significant similarity to *L. hilgardii* FLUB plasmids. Plasmid 1 of *L. hilgardii* FLUB, which is the largest of all those detected in this strain, is the most similar to the plasmid of *L. curvatus* WiKim38 (align length 1,940,170 bp), and plasmid 2 to that *of L. sakei* J64. Besides 22 genes of hypothetical proteins, it mostly contains genes encoding mobile element proteins (6), transposases (4) for copper-translocating P-type ATPase and cadmium-, zinc-, and mercury-transporting ATPase, and other uncharacterized transporters. Of some further interest, it has a DDI (DNA-damage-inducible) gene, a type of gene that is induced/expressed in response to stress, particularly genotoxic stress [41]. It has been shown that copper-translocating P-type ATPase is involved in resistance to copper [42]. An arsenate reductase-related protein, together with other genes (also chromosomally encoded), provides resistance to arsenic by discharging arsenite and antimonite. The genomic annotation indicates that *L. hilgardii* FLUB may be resistant to metals. Moreover, it contains a gene coding for the inner membrane protein YbiR, which is unique to this strain, and may derive from plasmid pL21533-3 (*Pediococcus damnosus*) or plasmid pLb464–8 (*L. brevis* BSO). Plasmid 2 of *L. hilgardii* FLUB is most similar to plasmid pWIKIM01 of *L. allii* strain WiKim39, isolated from scallion kimchi [43]. Besides genes encoding mobile element proteins (9), replication initiation protein A (2), and an LtrC-like protein (3), the genes that deserve the most attention in this plasmid are those associated with the detoxification of arsenic-containing substances: arsenate reductase (EC 1.20.4.4), arsenical resistance operon repressor, arsenite/antimonite pump-driving ATPase ArsA (EC 3.6.3.16), arsenite/antimonite:H+ antiporter ArsB, and asenic metallochaperone ArsD. The presence of the ArsB gene in the genome alone allows the bacteria to extrude As (III) from the cell via membrane potential [44]. When the ArsA gene is also present, the possibilities increase: together, ArsB and ArsA form a membrane-bound complex in which ArsB serves as a membrane-bound anchor and ArsA as an ATP-dependent pump for arsenite [45,46]. ArsD, as a metallochaperone, supports ArsA by transporting As (III) to the pump binding site, and is also responsible for the expression of the ars operon [47,48]. Research is currently underway to address this aspect. Interestingly, the FLUB chromosome also contains ArsR and ArsC genes, which further extend the strain’s arsenic resistance capabilities [49]. This may indicate horizontal transfer of other genes. Genes associated with arsenic in the FLUB genome offer the promising prospect of using these bacteria for the detoxification of arsenite. In the case of plasmid 3, of size 28,299 bp, alignment of this fragment is assigned to *L. plantarum* LMT1–48. In *L. hilgardii* FLUB, this plasmid encodes S-layer proteins, Sortase A, and a specific LPXTG domain (NCBI S-layer RefSeq: WP_013728337.1). The S-layer gene sequence (locus tag GQR93_RS15135) is probably a repeated, but altered, sequence of this chromosomally encoded protein (two matches, 738,944 to 739,797 and 747,055 to 747,762). The gene is also similar to the chromosomally encoded gene in LMG 07934 (Mauve alignment), but all indications are that it is unique to FLUB (singletons chapter). Plasmid 4 harbors genes of the bacteriocin helveticin, D-alanyl-D-alanine carboxypeptidase (EC 3.4.16.4), and replication initiation protein A predicted by PGfams. The gene identified as bacteriocin is unique to *L. hilgardii* FLUB, as is the D-alanyl-D-alanine carboxypeptidase gene. A comparison performed using PATRIC showed that the bacteriocin gene (PLF_1578_00042486) was similar to a gene found in the genome of *L. parafarraginis* DSM 1890. The last plasmid is the smallest of all; it carries three genes of an unknown function, a plasmid recombination enzyme gene, and genes specific for protein replication. Additionally, a heatmap was prepared, showing the presence of coding sequences for proteins classified to individual protein families as part of the FIGFam classification of individual plasmids of the *L. hilgardii* FLUB strain (supplement). It clearly shows that individual plasmids are characterized by very different content. The extraordinary resistance of *L. hilgardii* FLUB to adverse environmental conditions and its ability to adapt to such hostile environmental niches are probably increased by the presence of plasmids.

### 2.4. Pangenome of L. hilgardii Species

Since 2005, when Medini and others introduced the concept of the pangenome [50], there has been an increase in the number of studies based on this tool. In this article, an attempt was made to define the genomic homogeneity of the species *L. hilgardii*. The pangenome analysis approach was used to provide a genomic overview of the species and to highlight the novelty of the strain. In order to provide various dependencies for the analysis of the pangenome of the species *L. hilgardii*, orthologue detection was used, which allowed the identification of the core, accessory, and singleton genomes. The characteristics of the genomes of the five *L. hilgardii* strains available in the NCBI database are given in Table 2 (three records for the same strain are available, the genome with the highest level of completeness was taken for analysis). A list of genes, including pangenome subdivision and clusters of orthologous groups of proteins (COGs), for each strain is available in the Supplementary Materials, as well as an interactive Krona graph.

As a result of the analysis, 2059 genes (49.3%) were assigned to the core category, 1210 to the accessory category (28.9%), and 912 to singletons (21.8%). Despite the fact that only a few genomes of the species *L. hilgardii* have been sequenced and published, the sizes of the *L. hilgardii* core and total gene clusters are within the range of those of other species in the genus *Lactobacillus.* According to van Tattelin et al., the size of the core genome should be between 20–60% of the pangenome [51]. For the core genome of *L. hilgardii*, this ratio was 49.3%. The relatively small number of genomes included in the analysis has a positive impact on phylogenetic reliability. It is crucial that the phylogenetics of the strains selected for analysis be critically assessed. The fact that the ANI values are not lower than 97% excludes the possibility of an incorrect estimation of the pangenome. In attempting to provide a comprehensive insight into the evolution of bacterial pangenomes, one should limit the investigations to the following questions, because phylogenetic misclassification of a strain can lead to a drastic overestimate of the size of the species’ pangenome. Among the analyzed genomes, that of *L. hilgardii* FLUB is the largest, at 3.19 Mbp. There are small differences in the GC content, which ranges from 40.02% to 39.6%. This means that the selected strains represent a uniform taxonomic category. Notably, the number of genes encoding proteins is, again, the highest in *L. hilgardii* FLUB, which indicates that the bacteria have experienced a loss and/or acquired certain genes in order to adapt to the mead environment.

**Figure 5:**
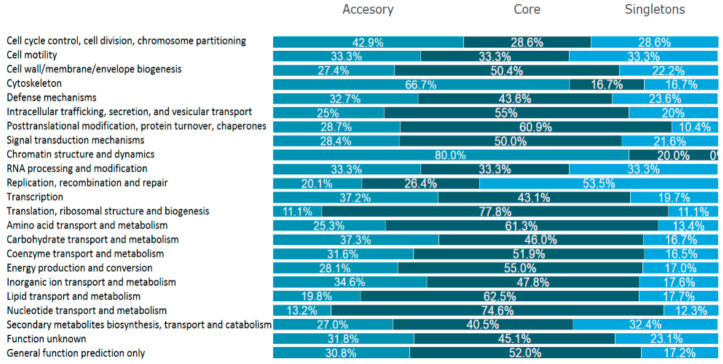
Distribution of clusters of orthologous groups of proteins (COGs) among accessory, core and singleton gene groups.

To illustrate their functionality, the proteins encoded by the sequenced genomes were combined into clusters of orthologous groups of proteins (COGs), which allowed us to compare the functional distribution among the three differentiated pangenome components (core, accessory, and singleton genomes). A total of 4181 coding sequences were assigned to four overarching groups: (1) cellular processes and signaling, with eight subgroups; (2) information storage and processing, with five subgroups, (3) metabolism, with eight subgroups; and (4) poorly characterized, with two subgroups. With regard to these four major groups, both the core and the accessory genomes have the largest proportions of genes related to metabolism, 35.5% and 39.3%, respectively; in singletons, this group represents 26% of all genes and is the second largest group after information storage and processing genes, which account for 32.6% of the strain-specific genome. As easily predicted, among the 23 subgroups, (S) function unknown and (R) general function prediction only are the most abundant in the core category. Apart from these poorly characterized groups, most of the core protein-encoding genes belong to the following functional subgroups: (E) amino acid transport and metabolism (225), followed by (J) translation, ribosomal structure and biogenesis (154), (K) transcription (125), and (G) carbohydrate transport and metabolism (163). These data are very coherent because the most numerous groups determine the high activity of the translational apparatus and thus the microbes’ ability to take advantage of different environmental conditions. In the genomes of other *lactobacilli*, most genes are members of the information storage and processing group. In the accessory section, the most numerous group, aside from the previously mentioned “poorly characterized”, are (G) carbohydrate transport and metabolism (132). Interestingly, most of the unique genes belong to the replication, recombination, and repair category (215 genes, which account for 23.6% of all singleton genes). Another numerous subgroup of singleton genes, apart from the poorly characterized ones, are carbohydrate transport and metabolism genes. When considering the concept of the pangenome in terms of the individual COG groups, it is worth mentioning that the individual strains share the most genes from the translation subgroup (J, 77.8%), but it can also be seen that each of the strains has an almost equal number of unique genes in the metabolism group. In general, this analysis shows that the species *L. hilgardii* has a high metabolic capacity as the genes that are the most prominent are those associated with genetic information and its processing, followed by genes that are involved in the transport and metabolism of carbohydrates and amino acids, regardless of whether they are core, accessory, or singleton genes (Figure 5). These COG categories are vital for the adaptation of *L. hilgardii* to an environment that requires tolerance to a high alcohol content, high acidity, and diluted nutrients [52,53].

**Figure 6:**
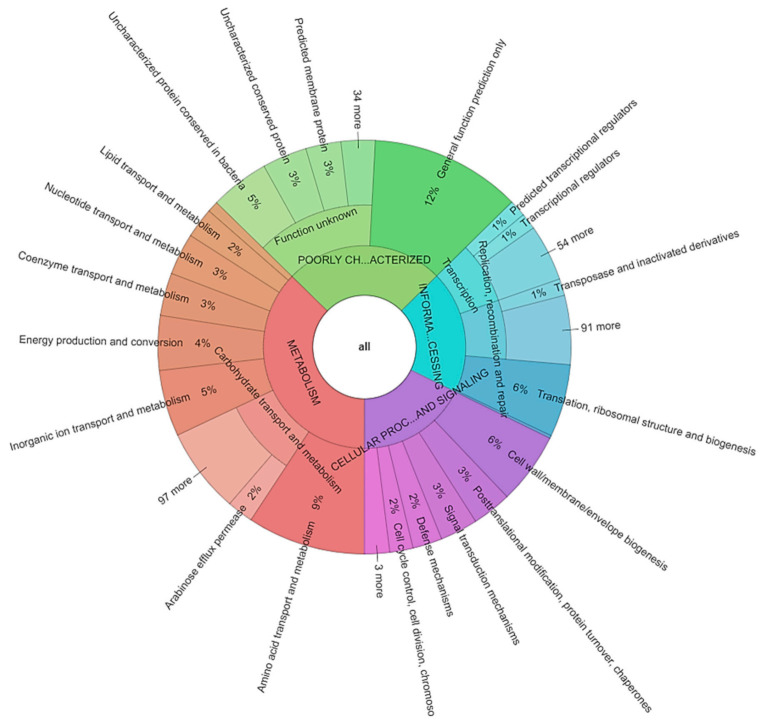
A snapshot of a Krona hierarchical data circle graph showing COG distribution in the complete sequence of *L. hilgardii* FLUB. A multi-layered interactive version with zoom and four-step depth adjustment is available via link or in the Supplementary Materials, as well as graphs for the individual replicons or pangenome.

As for *L. hilgardii* FLUB, the distribution of genes among the COG categories (Figure 6) reveals that most of the FLUB genes are in the “poorly characterized” category (S-function unknown, 422 genes, 13.5% of all genes, and R general function prediction only, 368 genes, 11.8%). The fact that these genes have no annotation confirms the novelty and unexplored potential of this strain. Other highly represented COG groups in the FLUB genome are transport and metabolism of amino acids (E, 289 genes, 9.3%) and carbohydrates (G, 272 genes, 8.7%). *L. hilgardii* FLUB carries 56 more category G genes than *L. hilgardii* DSMZ, which most differentiates the mead strain from the wine strain. Additionally, noteworthy with regard to adaptability is the cell wall/membrane/envelope biogenesis category (175 genes, 5.6%). In most bacteria, compounds present in alcoholic beverages (ethanol and sulfites, but also pH) violate membrane integrity, which is necessary to maintain homeostasis and energy activity [54]. Another difference between the strains FLUB and DSMZ is that the former has 29 more genes in COG category P “inorganic ion transport and metabolism” (162 genes, 5.2%). For example, the *ars* genes mentioned above are a unique feature of this strain. Having these genes may seem unusual, since arsenic is a common water pollutant, but since water is the main component of alcoholic beverages, this compound has also been detected in beers and wines [55].

### 2.5. Singletons of L. hilgardii FLUB

Out of the 4181 genes identified in the pangenome analysis, 912 were assigned as singletons, i.e., genes unique to the particular strains of the species *L. hilgardii* (Supplementary Materials provide a detailed list of genes). The largest numbers of strain-specific genes were found in the genomes of LMG 07934 (35.4%), FLUB (30.9%), MGYG (13.2%), LH500 (12.7%), and DMS (7.8%). A large number of genes classified in the singletons group are associated with the open character of the pangenome; with the arrival of new genomes from this species, the number of accessory genes also increases. From Roary output files, we extracted COG groups found only in the novel strain isolated from mead (Supplementary Materials). Of the 266 singleton genes identified in the genome of *L. hilgardii* FLUB, almost half (41%) were classified as hypothetical proteins (104) or uncharacterized proteins (6). An examination of this group of genes can be used to explore the unique metabolic abilities of this strain, often revealing a variety of lifestyles [56]. The survival of the FLUB strain in the mead environment depends on the bacteria’s ability to metabolize high concentrations of various sugars. The FLUB genome contains a number of genes related to sugar metabolism. One of them is a gene coding for a glycoside hydrolase family protein which is responsible for the breakdown of glycosidic bonds, i.e., the degradation of biomass components such as cellulose, hemicellulose, and starch. The FLUB genome also carries three repetitions of the alpha-N-arabinofuranosidase gene responsible for hemicellulose breakdown. Interestingly, this strain is extremely enriched in genes encoding glycoside hydrolase families (CAZy database): as many as 23 families were recorded, compared to only 9 families found in the related strains LH500 and LMG 07934. The FLUB genome carries glycoside-pentoside-hexuronide (GPH) transporter genes, not found in other *L. hilgardii* strains, which are responsible for the flow of sugars and which may contribute to the strain’s better adaptation to the high sugar concentrations in mead. In total, five unique carbohydrate transporter genes were detected in the FLUB genome: two GPH transporter genes, a major facilitator superfamily protein (YjmB), a di- and tricarboxylate transporter, and an Na/xyloside symporter-related transporter. Di- and tricarboxylates are semi-finished products of the tricarboxylic acid cycle (TCA). Bacteria with a carboxylate transporter in the membrane inside the cell use carboxylate as an additional source of energy under both aerobic and anaerobic conditions. Another significant group of strain-specific genes are those responsible for information storage and processing (highly represented in the pangenome analysis) such as the genes encoding replication proteins (10), transposases (36), integrase (3), and resolvase (1). Phage sequences are also represented in abundance (29). Several genes were classified as antimicrobial resistance genes: they included mecl_2 and mecl_3 genes of penicillin-binding protein 2 responsible for resistance to β-lactam antibiotics like methicillin, lytA autolysin acting as a virulence factor [57], a tree antibiotic efflux pump, macB (an ABC transporter), efmA_2 (an MFS transporter), and smeS (resistance-nodulation-cell division). The genetic variation between *L. hilgardii* strains is likely to have been caused by two processes, horizontal gene transfer and mutations, which appear to have been exacerbated in FLUB and LMG 07934 strains after they moved away from their common ancestor, leading to considerable alterations within this gene pool.

### 2.6. Carbohydrate Metabolism

The characteristics of carbohydrate metabolism in *L. hilgardii* FLUB were determined on the basis of pathway annotations and enzyme-degrading complex carbohydrates from these pathways. The COG functional categories of the genome of *L. hilgardii* FLUB are shown in Figure 6. This strain has 272 COGs involved in carbohydrate metabolism and transport, and 133 encoded proteins involved in energy production and conversion. It is worth noting that *L. hilgardii* FLUB has the highest percentage of carbohydrate transport and metabolism genes (8.7%) compared to the reference strain NRRL and to other *Lactobacillus* species [58,59]. The abundance of genes assigned to this category in this strain significantly increases its growth rate on different carbon sources. The reconstruction of the metabolic pathways revealed that *L. hilgardii* FLUB harbors metabolic pathways such as the tricarboxylic acid cycle (TCA), glyoxylate and dicarboxylate metabolism, butanoate metabolism, fructose and mannose metabolism, galactose metabolism, amino sugar and nucleotide sugar metabolism, the pentose phosphate pathway, pentose and glucuronate interconversions, ascorbate and aldarate metabolism, glycolysis/gluconeogenesis, pyruvate metabolism, propanoate metabolism, starch and sucrose metabolism, inositol phosphate metabolism, and C5-branched dibasic acid metabolism. Of these, the following are involved in central metabolism (RAST annotation): the TCA cycle, pyruvate metabolism, glycolysis, and the gluconeogenesis and glycolate pathway. The Embden–Meyerhof–Parnas pathway, considered to be the most widely distributed pathway of glucose metabolism, was also identified in the genome of *L hilgardii* FLUB based on the presence of genes encoding the enzymes glucose-6-phosphate isomerase EC 5.3.1.9 (three occurrences), pyruvate dehydrogenase EC 1.2.4.1. (two occurrences), and glucokinase EC 2.7.1.2 (two occurrences), and 13 other enzymes of this pathway. A comparison of heatmaps generated by PATRIC showed that *L. hilgardii* FLUB lacks succinate dehydrogenase, which occurs twice in the reference strain ATCC 8290 (Supplementary Materials). At the genomic level, *L. hilgardii* FLUB harbors transporters belonging to different transporter families, such as phosphotransferase system (PTS), transporters of various sugars (mannose, fructose, sorbose, lactose, cellobiose, and ribitol (11)), ATP-binding cassette (ABC) transporters of cobalt, zinc, sulfate, iron, phosphate, heme, methionine, glycine, betaine, proline, and choline, and many more. The genome of *L. hilgardii* FLUB also encodes glycoside-pentoside-hexuronide (GPT) cation symporter family transporters, oligosaccharide flippase, major facilitator superfamily (MFS) transporters (which not only take sugars, but also drugs and TCA cycle metabolites), and five H+ symporters for fructose, gluconate, and sodium [60].

### 2.7. Antimicrobial Resistance Genes

Many antibiotics occur naturally, and microbes inevitably develop resistance to them, as a result of interactions with the environment, in order to survive. The Genome Annotation Service in PATRIC is a powerful tool for determining antimicrobial resistance (AMR) genes [61]. We used PATRIC’s AMR classifier module to predict the antibiotic resistance of *L. hilgardii* FLUB, categorizing the resistance genes according to the following strategies (specific biochemical pathways): antibiotic target modifying enzyme, gene conferring resistance via absence, protein altering cell wall charge conferring antibiotic resistance, and antibiotic target in susceptible species (Table 3). Only one gene classified as an antibiotic target modifying enzyme, RlmA(II), was detected in *L. hilgardii* FLUB. This gene was revealed to be 100 amino acids shorter. When comparing to the other strains from the pangenome analysis, all the genes are mostly similar to the MGYG-HGUT-01333 strain, which aligns with the phylogenetic studies. The bacterial 23S rRNA methyltransferase encoded by this gene improves resistance to tylosin and tylosin-like macrolide antibiotics. It methylates the 23S ribosomal subunit located in a stem-loop, where the macrolide normally binds [62]. The gene is found in other Gram-positive bacteria, and is also present in *L. hilgardii* strains LH500 and LMG 07934. Another AMR mechanism detected in *L. hilgardii* FLUB is resistance conferred via absence of a gene. The absence of the gidB gene, encoding a 16S rRNA methyltransferase (EC 2.1.1.170), provides low-level resistance to aminoglycoside antibiotics, such as streptomycin [63]. It is believed that homologues of genes responsible for antibiotic resistance are involved in standard cell wall synthesis in LAB [22]. *L. hilgardii* FLUB harbors three genes encoding proteins altering cell wall charge conferring antibiotic resistance: GdpD glycerophosphoryl diester phosphodiesterase (EC 3.1.4.46), MprF L-O-lysylphosphatidylglycerol synthase (EC 2.3.2.3), and PgsA CDP-diacylglycerol-glycerol-3-phosphate 3-phosphatidyltransferase (EC 2.7.8.5). All of these genes confer resistance to daptomycin, and MprF also to defensin. Genes coding for antibiotic target proteins in susceptible species include Alr, Ddl, EF-G, EF-Tu, folA, Dfr, folP, gyrA, gyrB, inhA, fabI, Iso-tRNA, kasA, MurA, rpoB, rpoC, S10p, and S12p. *L. hilgardii* FLUB harbors changed gyrA and gyrB genes, which affect resistance to fluoroquinolone by changing the target site. An altered ddl gene (D-Ala-D-Ala ligase) changes the synthesis of peptidoglycan and may be responsible for resistance to cycloserine. Altered translation elongation factor G and Tu genes, also present in *L. hilgardii* LMG 07934 and LH500, may confer resistance to fusidic acid, kirromycin, enacyloxin IIa, and pulvomycin. A mutation in the rpoB and rpoC genes encoding the B and C subunits of RNA polymerase may be responsible for the acquisition of antibiotic resistance to rifamycin, daptomycin, rifabutin, and rifampin. *L. hilgardii* FLUB has a distinctive antibiotic resistance strategy related to its genetic background and therefore responds to selective pressures, which may influence the formation of resistance. All genes described above are chromosomally coded. A summary of the AMR genes annotated in the genome of *L. hilgardii* FLUB and corresponding AMR mechanisms is provided in the Supplementary Materials.

### 2.8. Osmoregulation, Detoxification, and Stress Response

The type and intensity of a given environmental factor shape the tolerance range of any bacteria wishing to occupy a given niche. However, it is impossible to determine which genes are responsible for the survival of bacteria in a particular environment, as this very often depends on many overlapping factors. This section describes several genes that may be responsible for the extraordinary adaptability of *L. hilgardii* FLUB to mead. Fourteen genes predicted in RAST, classified as “stress response” genes, were detected in the FLUB genome. A crucial aspect of survival in mead is the ability to adapt to the osmolarity of this environment, i.e., the high sugar and alcohol concentrations. *L. hilgardii* FLUB carries the following genes belonging to the choline and betaine uptake and betaine biosynthesis subsystem: proV, OpuAC, OpuAA, and OpuAB. These genes are responsible for the uptake and accumulation of osmoprotectants such as betaine, but also for the membrane transport of choline, which is necessary for the biosynthesis of glycine betaine. *L. hilgardii* FLUB appears to be capable of biosynthesizing glutathione, as it carries genes encoding the glutathione biosynthesis bifunctional protein gshAB (EC 6.3.2.2) (EC 6.3.2.3) and glutamate-cysteine ligase (EC 6.3.2.2). This has a number of interesting consequences, since these genes play an important role in various physiological processes. Among others, they provide a reduced cellular environment, with glutathione acting as an antioxidant protecting biomolecules, DNA, and proteins from oxidative damage [64]. The glutamate-cysteine ligase gene is chromosomally coded (GQR93_00790, GQR93_00800, GQR93_00810), while the gshAB gene is located on plasmid 1. This latter gene is, at the same time, also unique in terms of the concept of the pangenome of *L. hilgardii*. The plasmid gene is the most similar to the sequence found on plasmid 1 of *L. plantarum* strain SRCM103418, which was used to produce glutathione in reduced form [64]. These two genes encode enzymes which form a subpathway of glutathione synthesis from cysteine and glutamate. Additionally, the genome of *L. hilgardii* FLUB is equipped with 3-phosphoglycerate dehydrogenase genes (five repetitions) involved in the control of carbohydrate starvation.

### 2.9. Phenotypic Properties (Growth Characteristics, API-ZYM, SDS-PAGE)

**Figure 7:**
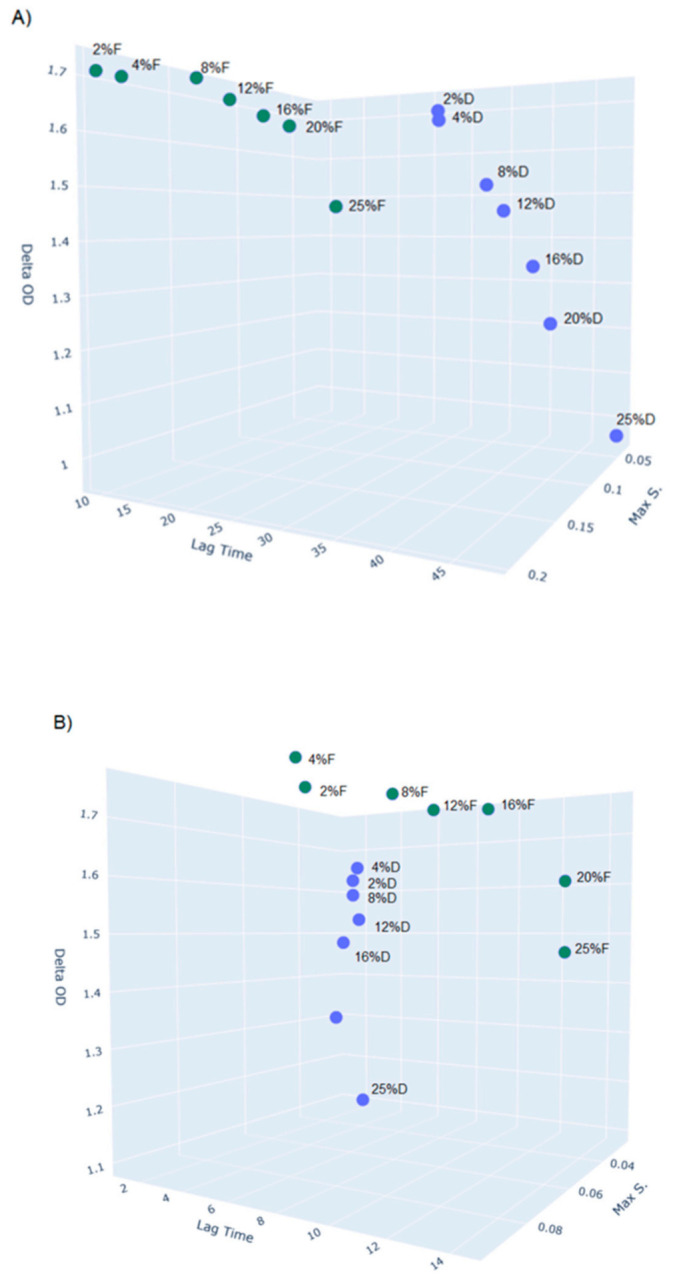
Growth kinetics of *L. hilgrardii* FLUB (green, F) and *L. hilgardii* DSMZ (blue, D) visualized as a three-dimensional scatter plot using plotly (Plotly Technologies INC., Montréal , QC, Canada): (**A**) growth kinetics on MRS medium (without dextrose) enriched with fructose in the concentration range of 2–25%, (**B**) growth on MRS supplemented with glucose (2–25%). An interactive version and parameters calculated according to Hoeflinger et al. are available in the Supplementary Materials [65].

This section focuses on selected experiments which illustrate the phenotypic differences between *L. hilgardii* FLUB, isolated from mead, and *L. hilgardii* DSMZ, isolated from port wine, stemming from their adaptation to the source of isolation. The strains’ preference for a primary source of carbon, tolerance to high concentrations of ethyl alcohol, metabolism of other carbohydrates, enzymatic profiles, and protein profiles were tested. A Bioscreen C analysis was performed to assess the growth preferences of *L. hilgardii* strains FLUB and DSMZ in relation to the concentration of glucose, fructose, ethanol, and various carbon sources in the growth medium. Data were analyzed using PYTHON according to Hoeflinger et al. [65] (Supplementary Materials) to calculate lag time, maximum specific growth rate, doubling time, delta OD, and other parameters. The media were selected to reflect the environmental conditions of wine or mead and were used to determine the strains’ growth limits at different concentrations of a given factor. Honey and port wine are known to have a high degree of sweetness, and both FLUB and DSMZ grew well on a sugar-enriched medium. Both strains were able to grow at all the glucose and fructose concentrations used (2–25%), however, there were significant differences visible due to the application of the growth kinetics (Figure 7).

As we focus in this article on the adaptation of the FLUB strain to the niche of mead, the 3D chart shows the full dynamics of this strain compared to the DSMZ strain isolated from port wine. A noticeable clustering of data for the two strains is visible. The 3D graph (A) shows that FLUB has a considerable advantage when growing on fructose. This is supported by the tree values used in plotting the graph. First of all, for the lag phase, there is a 16 h gap difference between the results set for FLUB (9–16 h) and DSMZ (32–48 h). The length of the lag phase reflects the possibilities of intracellular mechanisms to regulate metabolic processes. This grouping of data shows that, when inoculated onto fructose medium, the FLUB strain needs a relatively short time to adapt to its environment, thus the FLUB strain is “immediately” able to use fructose as its main carbon source. The major distinction between the two strains was that FLUB adapted to the 25% fructose concentration faster than DSMZ did to the 2% fructose concentration. Secondly, determinations of optical density, which is a measure of the ability of cells to multiply, showed that the growth of FLUB did not decrease so drastically with the increase in the concentration of fructose (max OD 1.7–1.5) as in the case of DSMZ (the lowest concentration of fructose 2%—max OD 1.66; the highest concentration of glucose—max OD of only 0.96). The last parameter considered was the max specific growth rate (1/hours), which is determined at the time when the cells are already adapted to the environment and multiply with a maximum capability. Here, also *L. hilgardii* FLUB achieved a higher value (0.1–0.2) than DSMZ (0.05–0.1). The sugar content of the sweetest mead (known as “gold” mead) can be as high as 20%, while its fructose content ranges between 60–240 g/L [66,67]. Although this article demonstrates that *L. hilgardii* FLUB has adapted to high concentrations of sugars corresponding to those found in mead, the most interesting feature of this strain is its preference for fructose over glucose, which is indicative of the fructophilicity of this strain. Fructophilic LAB are a relatively recently described group of bacteria [68], which do not form a phylogenetic cluster. Species belonging to this group exhibit the following characteristics: preference for fructose over glucose, elevated growth rate at high sugar concentrations [69], and a simple-sugar-rich source [70,71]. Some fructophilic strains are characterized by mannitol production [72,73], the presence of a bifunctional alcohol/acetaldehyde dehydrogenase gene [74], or/and lack of growth without an additional electron acceptor [75]. An analysis of post-culture fluids of FLUB carried out in a study which culminated in a patent application for this strain (P.429133) showed that the novel strain produced substantial amounts of mannitol. Considering the size of the genome of *L. hilgardii* FLUB, the functional analysis and the description of its unique genes, additionally supported by the phenotypic data, it can be concluded that the strain’s adaptation to the fructophilic environment of mead is most likely due to the acquisition of genes. This presents a different strategy than that observed in the genus *Fructobacillus*, in which adaptation to fructose led to the loss of certain genes, resulting in smaller genomes. 

**Figure 8:**
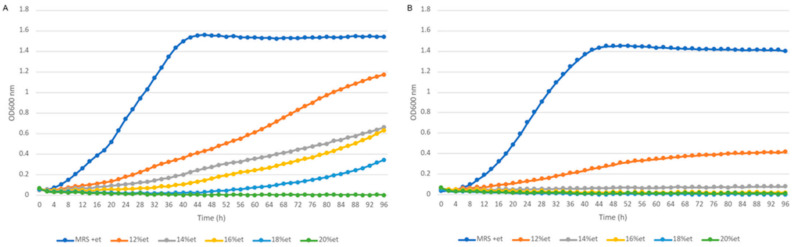
Growth curves of *L. hilgrardii* FLUB (**A**) and *L. hilgardii* DSMZ (**B**) on a medium supplemented with 12–20% ethanol, and control (MRS).

We also examined the growth of the strains FLUB and DSMZ on glucose, the principal source of energy. The lag phase for FLUB was found to be longer (8–14) than for DSMZ (1–8). There were no significant differences between the strains during growth at low glucose concentrations of 2–4%. At higher concentrations, however, the lag phase became longer for FLUB, as the glucose level in the medium increased. DSMZ showed a reverse trend, which demonstrates its adaptation to high glucose concentrations. The optical density for FLUB growing on glucose was practically the same as when fructose was used. DSMZ had the highest optical density when growing in a 4% glucose medium. These findings lead to the conclusion that FLUB, in general, has a greater ability to produce biomass than DSMZ. However, the two strains show no significant differences in the max specific growth rate. Environmentally unfavorable for the bacteria to live in is the presence of ethanol, which in mead varies up to 18%. Figure 8 provides the growth limits of *L. hilgardii* FLUB and DSMZ at 12, 14, 16, 18, and 20% concentrations of ethanol.

DSMZ could grow at ethanol concentrations of up to 12%, while FLUB was able to grow in a medium supplemented with 12–18% ethanol. No growth was achieved at the 20% ethanol concentration. Despite the fact that FLUB is generally more resistant to the adverse influence of ethanol, it requires more time to adjust to this substance. As lag time values show, FLUB needed 16 h, and DSMZ 10 h, to occupy the medium and initiate multiplication at a maximum rate.

The last element tested with the Bioscreen C system was the ability of *L. hilgardii* strains FLUB and DSMZ to use different carbon sources. Seventeen carbon sources were tested (apart from glucose and fructose). Both strains showed growth on glycerol, erythritol, saccharose, l-malic acid, amylose, xylose, lactose, galactose, arabinose, mannose, and rhamnose. Only FLUB could grow on ribose and starch, whereas DSMZ additionally metabolized mannitol. Therefore, the FLUB strain has a richer carbohydrate metabolism profile: it possesses more genes (272) in the COG category carbohydrate transport and metabolism than the reference strain (216). This can be explained by the different sugar profiles of mead and port wine, but also by the fact that the information provided in the genome of *L. hilgardii* FLUB allows these bacteria to exploit high concentrations of the most common sugars, glucose and fructose, more effectively than a wide profile of different sugars. Since FLUB is unable to metabolize mannitol, it is a potential producer of this polyol. In their research devoted to the development of phenotypic identification methods, Endo and Dicks found that the ability to ferment xylose distinguished *L. hilgardii* from *L. fructivorans* and *L. malefermentans* [53], and was probably due to the considerable amounts of xylose present in wines. In the case of *L. hilgardii* FLUB, the loss of the gene encoding xylose metabolism is favorable for its adaptation to mead, which does not contain large amounts of this sugar. It is worth mentioning that a gene coding for xylose isomerase (locus GQR93_01935) was detected in the FLUB genome, but this enzyme, in addition to catalyzing D-xylose conversion, is also known to be involved in the inter-conversion of fructose and glucose, which probably is further evidence for the strain’s specialization in the metabolism of these sugars. 

Another phenotypic experiment was carried out to compare the enzymatic profile of *L. hilgardii* FLUB to that of the reference strain DSMZ. The results of the API ZYM (Biomerieux) analysis of FLUB demonstrated the production of esterase (C4), esterase lipase (C8), lipase (C14), leucinearylamidase, valinearylamidase, cystine arylamidase, acid phosphatase, naphthol AS-BI-phosphohydrolase, β-galactosidase, α-glucosidase, α-galactosidase (three repetitions in the genome), β-glucosidase, *N*-acetyl-β-glucosaminidase, and β-glucuronidase (two repetitions in the genome). The strain had no alkaline phosphatase, trypsin, α-chymotrypsin, α-mannosidase, and α-fucosidase activities. The reference strain *L. hilgardii* DSMZ was similar to *L. hilgardii* FLUB in that it had genes encoding all the enzymes except β-glucuronidase, β-glucosidase, cystine arylamidase, and esterase (C4). The more complex enzyme profile of *L. hilgardii* FLUB indicates that this strain has a higher metabolic capacity than the reference strain, which is probably determined by the complex composition of carbohydrates contained in honey. Since the production of mead requires that the traditional technology be preserved, in the present study, we focused on compounds naturally occurring in mead or wine, and identified and compared the two strains’ preferences for these substrates, which are reflective of the strains’ sources of isolation. Knowledge of their growth preferences can be used in the future to develop methods of inhibiting the undesirable growth of these bacteria.

Because some authors propose that the tolerance of the species *L. hilgardii* to adverse ecological conditions is, to some extent, due to the occurrence of a complex set of surface proteins, we therefore tested the electrophoretic protein profiles of the FLUB and DSMZ strains. An analysis of the samples imaged on SDS-PAGE electrophoresis gels showed that FLUB had a novel protein profile (Supplementary Materials). The gel image demonstrated that FLUB had several protein fractions differing in size and accumulation from those of DSMZ, especially the fraction of about 68 kDa which was only visible in FLUB. We wanted to examine the profile of surface proteins of FLUB because the genes associated with the construction of the cell wall/membrane/envelope stand out quantitatively and qualitatively in the genome of this strain. They form an extensive COG group representing 5.6% of the genome and include strain-specific genes and/or genes appearing on plasmids. The genomic analysis also showed that the genome of *L. hilgardii* FLUB contains D-alanyl-lipoteichoic acid biosynthesis protein DltB (locus tag GQR93_02065) as well as several cell wall surface proteins, sortase (encoded chromosomally and on plasmid 3), two genes of the LPxTG cell wall anchor domain (GQR93_10305 and GQR93_12475), and many more. These genes may confer the ability to autoaggregate [76]. SDS-PAGE photos provide clear evidence of the changes that have occurred in the protein profile of this strain, compared to the reference strain. It can be concluded that *L. hilgardii* FLUB is much more resistant to adverse environmental conditions than the reference strain due to the presence of outer surface proteins. Because the outer protein layer constantly interacts with the surrounding environment, the surface proteins have acquired a function that ensures the survival of the bacteria in adverse environmental conditions. In their study, Dohm et al. found that the S-layer played an important role in the survival of *L. hilgardii* B706 in the harsh environment of wine [77]. This was confirmed by an examination of a culture of this strain in the stress conditions that may occur in wine, i.e., a low pH and the presence of alcohol, phenolic compounds, sulfites, and heavy metals.

## 3. Materials and Methods

### 3.1. Isolation and Identification

Microbiological samples were collected from the mead production line using sterile swabs in several variants and repetitions. Samples were placed in MRS (de Man, Rogosa and Sharpe) broth and incubated at 28 °C for 48 h in anaerobic and aerobic conditions. Then, the bacteria were purified by repeated streaking on MRS agar plates. Colonies were selected for further studies according to their characteristic shape, size, or color. The purity of the colonies was additionally checked by microscopic observations. The MALDI-TOF Biotyper (Bruker Daltonics, Bremen, Germany) method was used for initial identification. The analysis was carried out using the procedure described in detail in our previous paper [78]. Only one isolate was identified as a representative of the genus *Lactobacillus*, and it was this strain that was used in further studies. To confirm the affiliation of the *Lactobacillus* strain, 16S rRNA sequencing was performed [79]. Genetic material was isolated using a Genomic Mini AX Bacteria Spin kit (A&A Biotechnology, Gdynia, Poland) according to the manufacturer’s protocol. Primers (27f) 5′-AGAGTTTGATCCTGGCTCAG-3′ and (1495r) 5′-CTACGGCTACCTTGTTACGA-3′ (Genomed S.A., Warszawa, Poland) as well as PCR Master Mix (2×) (Thermo Fisher Scientific, Bremen, Germany) were used for the PCR reaction. The sequence was assembled using a DNA Baser Sequence Assembler and aligned with BLAST. The strain was maintained at −80 °C in MRS (Merck KGaA, Darmstadt, Germany) supplemented with 20% glycerol as stock collection. Prior to the analysis, the strain tested was regularly transferred into fresh sterile broth MRS and incubated (30 °C/24 h) in aerobic conditions.

### 3.2. Whole Genome Sequencing

To unravel the genomic properties of the *L. hilgardii* strain, whole genome sequencing (WGS) was carried out. Bacterial total genomic DNA was isolated using the CTAB/lysozyme method with the addition of the cell wall degrading enzymes lysozyme (20 mg/mL; Sigma-Aldrich, Dorset, UK) and mutanolysin (5 U/mL; A&A Biotechnology, Gdańsk, Poland). The quality of the DNA was tested on standard 0.8% agarose gel and 1% PFGE gel using a Biorad CHEF-III apparatus, and DNA template quantity was measured using a Qubit fluorometer. Genomic bacterial DNA was mechanically sheared to an appropriate size and used to construct paired-end TruSeq-like libraries utilizing a KAPA Library preparation kit (KAPA/Roche, Basel, Switzerland) following the manufacturer’s instructions. The bacterial genome was sequenced in the paired-end mode (v3, 600 cycle chemistry kit) using a MiSeq instrument (Illumina, San Diego, CA, USA). Illumina sequencing yielded 1,906,340 reads and 564,597,125 nucleotides of sequence data. The obtained sequence reads were filtered by quality using the FastX toolkit (http://hannonlab.cshl.edu/fastx_toolkit/, accessed on 7 March 2019), and residual Illumina adapters were removed using Cutadapt45. Quality-filtered Illumina data (1,204,850 paired reads and 357,606,583 nucleotides of sequence data) was assembled using Newbler v3.0 software (Roche, CT, USA) and Spades v3.11.1 (http://cab.spbu.ru/software/spades/, accessed on 7 March 2019) [80] to estimate the approximate size of the draft bacterial genome. In the next stage, long reads were generated using a MinION nanopore sequencing instrument (Oxford Nanopore Technologies, Oxford, UK). Bacterial DNA was sheared into approximately 20 kb fragments using a Covaris gTube device (Covaris, Ltd., Brighton, UK), and the library was prepared using an ONT 1D ligation sequencing kit (SQK-LSK108) with a native barcoding expansion kit (EXP-NBD103). Nanopore sequencing was performed using the NC_48 h_Sequencing_Run_FLO-MIN106_SQK-LSK108 protocol and an R9.4 MinION flowcell. Raw nanopore data was basecalled using Albacore v2.1.7 (Oxford Nanopore Technologies, Oxford, UK). After quality filtering and sequencing adapter removal using Porechop (https://github.com/rrwick/Porechop, accessed on 7 March 2019), 64,423 barcoded reads remained. The median read length of the dataset obtained was 10,745 nucleotides and 758,359,419 total bases. Long nanopore reads were assembled in a hybrid mode with the Illumina data using Unicycler v.0.4.447. Genome hybrid assembly resulted in six circular replicons: a 3 Mb size chromosome and five plasmids. The remaining sequence errors in genome assembly were verified by PCR amplification of the DNA fragments, followed by Sanger sequencing with an ABI3730xl Genetic Analyzer (Life Technologies, Carlsbad, CA, USA) using BigDye Terminator Mix v3.1 chemistry (Life Technologies, Carlsbad, CA, USA). All of the sequence errors and misassemblies were further corrected using Seqman software (DNAStar, Madison, WI, USA) to obtain a complete nucleotide sequence of the bacterial genome. The whole genome sequence was deposited as a BioProject (PRJNA595831) in GenBank under the accession number CP047121.1 (3,071,102 bp chromosome; plasmids CP047122.1 42,732 bp; CP047123.1 37,669 bp; CP047124.1 28,299 bp; CP047125.1 6896 bp; CP047126.1 3528 bp).

### 3.3. Annotation and Functional Categorization

Prokka 1.14.6 was the primary tool used to annotate the sequenced genome [81]. Additionally, PATRIC 3.6.5 [82], which utilizes the RASTtk tool [83] and the NCBI Prokaryotic Genome Annotation Pipeline (PGAP) [84], were employed for the same purpose as part of the submission pipeline, and the results were combined for further analysis. Annotation included estimation of the number of tRNA and rRNA sequences and CRISPR repeats. Circular maps for the particular replicons were generated using CGView [5]. Further functional analysis and clustering were performed using the reCOGnizer tool (https://github.com/iquasere/reCOGnizer, accessed on 29 July 2020) clusters of orthologous groups (COG) category for each CDS. COG assignments were visualized using Krona [85]. The genome of *L. hilgardii* was also annotated using the DDBJ Fast Annotation and Submission Tool (DFAST, https://dfast.nig.ac.jp, accessed on 20 May 2020), which is suitable for genomic analysis of LAB [86].

### 3.4. Phylogenetic Analysis

Next, the complete genome sequence was analyzed using the Similar Genome Finder service on the PATRIC portal, which uses MinHash [20] to find similar public genomes in PATRIC and compute genome distance estimation. For comparison, a PGAP pipeline taxonomy check was performed, which utilizes average nucleotide identity (ANI), to compare the input genome sequence to the genomes of the type strains in GenBank [4]. Type (Strain) Genome Server (TYGS) was used to determine the affiliation of the strain within the latest established phylogenetic species boundaries [87]. A method based on Genome BLAST Distance Phylogeny (GBDP) for whole genomes and 16 S, with digital DNA-DNA hybridization (dDDH) values from intergenomic distances was applied [87]. Pairwise comparisons of the new strain and *L. hilgardii* NRRL, i.e., ANI and correlation indexes of tetra-nucleotide signatures were performed using JSpeciesWS [19].

A phylogenetic tree was generated for 50 previously selected closely related genomes using the PATRIC Codon Tree service, which uses amino acid and nucleotide sequences from a defined number of PATRIC’s global protein families [32]. The protein families, known as PGFams, were randomly picked to build an alignment and then to generate a tree based on the differences between the selected sequences using the RAxML program [88]. The phylogenetic tree was visualized using iTOL [18].

### 3.5. L. hilgardii Pan-Core Genome

Complete genomic sequences of four *L. hilgardii* strains available in GenBank (one genome of strain DSMZ 20,176 was selected, which had the best possible assembly) were downloaded and analyzed together with *L. hilgardii* FLUB using Roary [89], applying standard parameters. The numbers of pan, core, accessory, and unique gene clusters were calculated from the Roary output files as well as a presence/absence table and a pan-core genome development chart. For all respective gene clusters, a representative sequence was obtained and assigned to individual COG groups using reCOGnizer, as described above. COG clustering was performed and visualized for core, pan, accessory, and unique genomes of all *L. hilgardii* strains analyzed. Additionally, for a whole genome comparison of all available *L. hilgardii* sequences and sequences FLUB strain, the genomes were aligned using Mauve 2.3.1 with the progressive Mauve algorithm utilized [90].

### 3.6. Phenotypic Properties

The phenotypic properties of the new strain were determined using a carbohydrate fermentation kit (HiCarbo; HiMedia, Mumbai, India) and API ZYM (bioMérieux SA, Marcy l’Etoile, France). In the carbohydrate fermentation test, the optical density was set to 0.5 at 600 nm in a 24 h culture, and then the inoculum was added to 35 wells containing different carbohydrates. The plates were incubated at 37 °C for 24 h. In the API ZYM test, OD was set to 0.8, and the inoculum was added to 20 wells. The results were read after 4 h incubation at 37 °C. The growth limits of *L. hilgardii* FLUB and *L. hilgardii* NRRL B-1843 were determined using the Bioscreen C system (Labsystem, Helsinki, Finland). Bioscreen C was used to perform three different research tasks: to test the strains’ preference for the main carbon source, to examine its adaptation to high concentrations of ethanol, and to investigate its ability to metabolize different sugars. After 24 h incubation, the bacterial cultures were centrifuged and removed from the medium. The bacterial cells were suspended in physiological saline, and optical density was again set to 0.5 at 600 nm. Cells in the log phase were used for the experiments. The bacteria were grown in MRS (or MRS without dextrose) supplemented with (1) glucose (2–25%) and fructose (2–25%), (2) ethyl alcohol (12–20%), and (3) various carbon sources. The sugar metabolism experiments were conducted using MRS medium without glucose supplemented with 2% of the following sugars: xylitol, erythritol, glycerol, starch, ribose, galactose, lactose, mannose, mannitol, rhamnose, arabinose, amylose, maltose, L-malic acid, saccharose, xylose, and also mixtures such as corn syrup, glucose-fructose syrup, and a mix of 2% glucose and 2% fructose. In triplicate, 350 μL of the media were transferred onto 100-well honeycomb plates, and the wells were inoculated with 50 μL of the bacterial suspension. The experiments were performed in aerobic and anaerobic conditions by measuring the OD at 600 nm every 2 h for 48 h. The growth plots were visualized using Plotly (https://plotly.com/, accessed on 12 August 2020). To characterize the new strain *L. hilgardii* FLUB, we also tested its protein profile with SDS-PAGE as surface proteins are crucial to survival in the harsh environmental conditions of alcoholic beverages. Protein profiles and outer surface proteins were isolated according to Waśko et al. [91] and visualized using SDS-PAGE.

## 4. Conclusions

In this paper, we report the whole-genome sequence of a novel strain, *L. hilgardii* FLUB, isolated from mead, an environment not previously colonized by LAB. The assembled genome consists of six contigs, one chromosome, and five plasmids, with a total length of 3,190,226 bp and an average G+C content of 40.09%. The published genome is the largest of the five genomes representing the species *L. hilgardii*, and one of the three publicly available genomes of this species with a complete assembly level. The assembled genome was submitted to the comprehensive genome analysis services, as a result, the PATRIC, RAST, and then COG, and GO annotations were obtained, which provided a comprehensive perspective on the lifestyle of *L. hilgardii*. One element that is part of the overall features ensuring the survival of the strain in the unfavorable environment of mead is the possession of a greater number of plasmids. All plasmids of this strain contain plasmid replication initiation proteins, which shows that they not only have a basic replicon, but also structures responsible for plasmid maintenance. Apart from a backbone, four of the five plasmids also contained multiple, mostly strain-specific, genes which act as additional “equipment”, increasing the amount of genetic information carried by the plasmids. One example of such “auxiliary” genes are genes that encode cell wall/membrane proteins, which are crucial for interactions with the external environment. They include genes that encode enzymes and transporters which increase *L. hilgardii* FLUB’s metabolic capabilities, genes that encode resistance to heavy metals, and genes responsible for antimicrobial resistance, allowing the strain to grow in honey, which is known for its bactericidal properties. The present article demonstrates experimentally that the genome of *L. hilgardii* FLUB contains biotechnologically interesting genes, such as the 23 glycoside hydrolase families, or the rich gene pool of the cell wall/membrane category with the unique plasmid (3) encoded genes Sortase A and LPXTG domain, and finally the ability of the strain to produce mannitol The growth kinetics experiments show that *L. hilgardii* FLUB demonstrates affinity for fructose in a two-pronged manner: it not only prefers fructose as a primary carbon source but can also grow in high concentrations of this sugar. This finding leads to the conclusion that an environment rich in fructose promotes the evolution of “fructophilicity” in bacteria, which present two different strategies in adapting to fructose-rich environments: the acquisition of genes, as in the case of *L. hilgardii* FLUB, or the loss of genes, as in the genus *Fructobacillus*. *L. hilgardii* FLUB poses a new threat to the mead industry, which is further complicated by the fact that the removal of the spoilage bacterium from the mead should not reduce the quality of the final product. In the present article, we characterized this novel, extremely osmo-resistant strain and examined its growth preferences and growth limits, the knowledge of which is key to the development of methods of eliminating this strain from mead.

## Figures and Tables

**Table 1:** General genome features.

	Chromosome	Plasmid 1	Plasmid 2	Plasmid 3	Plasmid 4	Plasmid 5	Total
Total Sequence Length (bp):	3,071,102	42,732	37,669	28,299	6896	3528	3,190,226
Number of Sequences:	1	1	1	1	1	1	6
Longest Sequences (bp):	3,071,102	42,732	37,669	28,299	6896	3528	3,071,102
N50 (bp):	3,071,102	42,732	37,669	28,299	6896	3528	3,071,102
Gap Ratio (%):	0.000000	0.000000	0.000000	0.000000	0.000000	0.000000	0.000000
CG Content (%):	40.12	39.44	41.56	37.09	35.64	37.39	40.1
Number of CDSs:	2858	50	41	30	6	4	2997
Average Protein Length:	303.2	225.3	226.5	156.9	162.3	206.8	298.4
Coding Ratio (%):	84.7	79.1	74.0	49.9	42.4	70.3	84.1
Pseudogenes	61	9	5	13	4	1	93
Number of rRNAs:	15	0	0	0	0	0	15
Number of tRNAs:	61	0	0	0	0	0	61
Number of CRISPRs:	1	0	1	0	1	0	3

**Table 2:** Genomes of *L. hilgardii* used for pangenome analysis. Data obtained from the NCBI Genbank database.

No.	Organism Name	Strain	BioSample	BioProject	Assembly	Assembly Level	Size (Mbp)	GC %	Scaffold	CDS	Number of Genes	Pseudogenes	rRNA	tRNA	Other RNA
1	*L. hilgardii*	FLUB	SAMN13567894	PRJNA595831	GCA_009832765.1	Complete	3.19	40.07	6	2871	3043	93	15	61	3
2	*L. hilgardii*	LMG 07934	SAMN14262734	PRJNA609644	GCA_011765585.1	Complete	2.77	39.7	1	2540	2739	12	15	61	3
3	*L. hilgardii*	LH500	SAMN12777270	PRJNA566016	GCA_008694025.1	Complete	2.65	39.8	1	2368	2603	157	15	60	3
4	*L. hilgardii*	MGYG-HGUT-01333	SAMEA5850835	PRJEB33885	GCA_902374015.1	Scaffold	3.14	40.2	106	2768	2930	99	3	57	3
5	*L. hilgardii*	DSM 20176	SAMN02369502	PRJNA222257	GCA_001434655.1	Contig	2.6	39.6	125	2387	2593	143	7	53	3
		ATCC 8290	SAMN00001467	PRJNA31489	GCA_000159315.1	Scaffold	2.72	39.9	113	2395	2615	158	3	56	3
		ATCC 8290	SAMN08557741	PRJNA434413	GCA_004354795.1	Scaffold	2.77	39.9	92	2523	2746	146	16	58	3

**Table 3:** Antimicrobial resistance (AMR) genes according to the Genome Annotation Service in PATRIC.

AMR Mechanism	Genes
Antibiotic target in susceptible species	Alr, Ddl, EF-G, EF-Tu, folA, Dfr, folP, gyrA, gyrB, inhA, fabI, Iso-tRNA, kasA, MurA, rpoB, rpoC, S10p, S12p
Antibiotic target modifying enzyme	RlmA(II)
Gene conferring resistance via absence	gidB
Protein altering cell wall charge conferring antibiotic resistance	GdpD, MprF, PgsA

## Data Availability

Not applicable.

## Supplementary Materials

The following are available online at https://www.mdpi.com/article/10.3390/ijms22073780/s1.
