# Supplementary material for: Genome and Pangenome Analysis of Lactobacillus hilgardii FLUB—A New Strain Isolated from Mead
Source: Int J Mol Sci. 2021 Apr 6;22(7):3780. doi: 10.3390/ijms22073780 (PMC8038741; doi:10.3390/ijms22073780)
Supplement: Supplementary file 1 [file ijms-22-03780-s001.zip › Supplementary Materials/Interactive charts/Growth plots Bioscreen/Plotly Figure 7A.html]

 


plotly-logomark
