# Supplementary material for: Genome and Pangenome Analysis of Lactobacillus hilgardii FLUB—A New Strain Isolated from Mead
Source: Int J Mol Sci. 2021 Apr 6;22(7):3780. doi: 10.3390/ijms22073780 (PMC8038741; doi:10.3390/ijms22073780)
Supplement: Supplementary file 1 [file ijms-22-03780-s001.zip › Supplementary Materials/Interactive charts/Krona COG/Krona_L.hilgardii_COG_chromosome.html]

Javascript must be enabled to view this page.

magnitude
magnitudeUnassigned

krona

2973

1130

264

1

1

1

1

6

6

1

1

3

3

4

4

1

1

2

2

1

1

3

3

5

5

23

23

2

2

3

3

3

3

1

1

1

1

1

1

1

1

1

1

1

1

1

1

1

1

3

3

2

2

2

2

1

1

2

2

1

1

5

5

1

1

2

2

2

2

61

61

3

3

1

1

2

2

1

1

1

1

1

1

2

2

1

1

1

1

1

1

1

1

2

2

3

3

1

1

1

1

1

1

1

1

2

2

6

6

2

1

1

1

1

2

2

1

1

1

1

1

1

1

1

1

1

4

4

1

1

2

2

4

4

1

1

1

1

1

1

1

1

1

1

2

2

1

1

1

1

1

1

4

4

2

2

1

1

1

1

1

1

1

1

1

1

5

5

2

2

2

2

3

3

3

3

3

3

2

2

2

2

1

1

1

1

8

8

2

2

4

4

1

1

1

1

1

1

101

1

1

1

1

2

2

2

2

1

1

1

1

1

1

1

1

1

1

1

1

2

2

1

1

1

1

1

1

2

2

1

1

1

1

1

1

2

2

1

1

2

2

2

2

2

2

1

1

4

4

2

2

1

1

1

1

1

1

1

1

4

4

1

1

1

1

1

1

1

1

1

1

1

1

3

3

1

1

1

1

1

1

1

1

2

2

3

3

1

1

1

1

1

1

2

2

2

2

4

4

2

2

3

3

2

2

1

1

2

2

2

2

1

1

2

2

1

1

3

3

1

1

1

1

1

1

1

1

2

2

1

1

22

1

1

1

1

1

1

1

1

3

3

1

1

1

1

1

1

1

1

1

1

1

1

1

1

1

1

1

1

1

1

1

1

1

1

1

1

1

1

1

1

102

1

1

1

1

1

1

1

1

1

1

3

3

4

4

1

1

2

2

2

2

1

1

1

1

1

1

1

1

1

1

1

1

8

8

1

1

1

1

1

1

1

1

1

1

1

1

1

1

1

1

1

1

4

4

1

1

2

2

1

1

1

1

1

1

2

2

1

1

1

1

1

1

1

1

1

1

1

1

2

2

1

1

1

1

1

1

1

1

1

1

1

1

1

1

1

1

1

1

4

4

1

1

4

4

1

1

1

1

2

2

1

1

1

1

7

7

1

1

1

1

2

2

1

1

1

1

2

2

2

2

1

1

149

1

1

2

2

1

1

5

3

2

1

1

1

1

1

1

1

1

3

3

2

2

1

1

1

1

5

5

1

1

1

1

1

1

1

1

3

3

4

4

1

1

1

1

2

2

2

2

1

1

1

1

2

2

4

4

2

2

1

1

2

2

2

2

2

2

2

2

1

1

2

2

3

3

1

1

1

1

1

1

1

1

2

2

2

2

4

4

2

2

1

1

14

6

8

2

2

1

1

3

3

3

3

4

4

1

1

6

6

1

1

1

1

1

1

1

1

1

1

1

1

3

3

1

1

3

3

3

3

2

2

4

1

3

1

1

1

1

1

1

1

1

3

3

1

1

1

1

2

2

74

1

1

1

1

1

1

1

1

1

1

1

1

5

5

1

1

1

1

2

2

1

1

2

2

1

1

2

2

1

1

1

1

18

18

1

1

1

1

1

1

2

2

1

1

1

1

1

1

1

1

3

3

1

1

1

1

1

1

1

1

1

1

5

5

2

2

1

1

1

1

2

2

1

1

1

1

2

2

1

1

288

1

1

2

2

1

1

1

1

1

1

1

1

4

4

1

1

2

2

6

6

5

5

1

1

1

1

1

1

6

6

1

1

1

1

6

6

4

4

2

2

2

2

1

1

1

1

1

1

1

1

3

3

1

1

2

2

3

3

1

1

1

1

1

1

1

1

1

1

1

1

1

1

1

1

1

1

2

2

1

1

2

2

8

8

1

1

1

1

1

1

2

2

12

12

2

2

1

1

1

1

9

9

1

1

1

1

1

1

1

1

1

1

2

2

1

1

6

6

1

1

1

1

1

1

1

1

6

6

1

1

1

1

1

1

1

1

1

1

2

2

3

3

1

1

2

2

3

3

1

1

9

9

3

3

4

4

1

1

2

2

2

2

2

2

1

1

1

1

1

1

1

1

1

1

5

5

1

1

3

3

1

1

3

3

1

1

2

2

1

1

3

3

1

1

1

1

4

4

1

1

2

2

1

1

1

1

3

3

17

2

15

1

1

4

4

1

1

1

1

2

1

1

2

2

1

1

1

1

1

1

2

2

1

1

2

2

1

1

1

1

1

1

1

1

3

3

4

4

1

1

1

1

2

2

1

1

1

1

1

1

2

2

3

3

7

7

1

1

1

1

1

1

1

1

130

4

4

4

4

1

1

7

7

1

1

1

1

1

1

2

2

2

2

1

1

3

3

1

1

1

1

1

1

2

2

3

3

1

1

1

1

1

1

3

3

2

2

1

1

9

9

1

1

1

1

5

5

1

1

1

1

2

2

1

1

1

1

1

1

2

2

1

1

1

1

4

4

1

1

1

1

1

1

2

2

1

1

8

8

1

1

1

1

2

2

3

3

1

1

2

2

1

1

1

1

2

2

1

1

1

1

2

2

1

1

1

1

1

1

1

1

2

2

1

1

1

1

1

1

2

2

1

1

1

1

2

2

2

2

4

4

1

1

1

1

524

24

1

1

1

1

3

3

1

1

1

1

1

1

1

1

2

2

2

2

2

2

1

1

1

1

1

1

4

4

1

1

1

1

95

1

1

1

1

1

1

1

1

1

1

1

1

1

1

3

2

1

1

1

10

10

1

1

1

1

1

1

2

2

3

3

1

1

1

1

2

2

1

1

1

1

1

1

1

1

1

1

4

2

2

2

2

1

1

1

1

2

2

2

2

1

1

2

2

1

1

1

1

2

2

2

2

1

1

2

2

2

2

1

1

1

1

4

4

2

2

2

2

1

1

1

1

1

1

2

2

4

3

1

1

1

2

1

1

1

1

1

1

1

1

1

1

1

1

1

1

1

1

1

1

1

1

31

1

1

1

1

1

1

1

1

2

2

1

1

1

1

2

2

2

2

1

1

1

1

2

2

1

1

1

1

1

1

1

1

1

1

1

1

1

1

1

1

1

1

1

1

2

1

1

3

3

165

3

3

4

4

2

2

1

1

1

1

1

1

1

1

1

1

4

4

1

1

3

3

1

1

1

1

7

7

3

3

1

1

2

2

1

1

2

2

1

1

6

6

1

1

4

4

1

1

2

2

2

2

7

2

5

2

2

2

2

2

2

1

1

2

2

1

1

1

1

1

1

5

2

3

2

2

1

1

1

1

4

4

2

2

1

1

2

1

1

2

2

1

1

1

1

4

4

1

1

1

1

3

3

1

1

2

2

1

1

2

2

2

2

3

3

1

1

8

8

1

1

1

1

1

1

5

5

1

1

1

1

1

1

2

2

1

1

2

2

1

1

3

3

2

2

6

6

1

1

2

2

1

1

1

1

1

1

3

3

1

1

3

1

1

1

1

1

1

52

1

1

1

1

1

1

1

1

2

2

1

1

1

1

2

2

1

1

2

2

2

2

2

2

1

1

1

1

1

1

3

2

1

1

1

15

15

1

1

1

1

1

1

2

2

1

1

1

1

4

4

2

2

82

1

1

1

1

1

1

2

2

2

2

1

1

2

2

2

2

2

2

9

9

2

2

2

2

1

1

4

4

1

1

2

2

1

1

1

1

1

1

1

1

1

1

4

4

4

4

2

2

13

2

2

9

5

5

1

1

8

8

1

1

1

1

1

1

2

2

72

1

1

2

2

1

1

3

3

1

1

1

1

7

7

4

4

3

3

1

1

7

7

1

1

1

1

9

9

15

15

2

2

1

1

1

1

2

2

5

5

1

1

3

1

2

763

402

6

6

3

3

2

2

1

1

5

2

2

1

1

1

1

1

1

1

1

1

2

2

1

1

2

1

1

80

2

1

1

1

3

1

4

1

6

1

2

4

1

1

1

1

1

1

6

2

1

2

2

2

1

5

1

4

1

1

1

3

1

1

2

2

1

1

3

1

1

1

1

1

1

1

1

14

1

1

1

5

2

1

1

1

1

1

1

8

1

1

1

1

1

1

1

1

10

10

2

2

2

1

1

1

1

1

1

2

2

1

1

2

2

1

1

1

1

1

1

137

1

1

1

1

1

1

1

4

1

1

5

2

2

1

1

1

1

1

1

1

1

1

1

1

1

1

2

1

1

2

3

1

1

1

1

1

1

2

1

1

1

2

1

1

1

1

1

1

1

1

1

1

1

1

2

1

1

1

1

1

1

5

2

1

2

1

1

1

1

1

1

1

1

1

1

1

1

1

1

1

1

1

1

2

1

2

1

1

1

1

1

1

1

1

4

1

2

1

1

1

1

1

2

1

4

2

2

1

1

1

1

1

1

103

1

1

1

1

1

1

1

1

1

1

1

6

2

1

1

1

1

1

1

1

1

1

1

2

1

1

1

4

1

2

1

4

1

1

1

1

1

1

1

1

1

1

1

1

1

1

2

1

1

1

1

1

1

1

2

3

1

1

1

1

1

1

1

1

1

3

1

1

1

1

1

1

1

2

2

1

2

1

1

1

2

2

361

1

1

1

1

1

1

1

1

2

2

4

4

1

1

8

8

3

3

1

1

1

1

4

4

1

1

2

1

1

1

1

1

1

2

2

1

1

4

4

1

1

1

1

1

1

4

4

6

6

1

1

1

1

1

1

1

1

2

2

1

1

1

1

1

1

1

1

2

2

2

2

1

1

1

1

1

1

3

3

4

4

2

2

1

1

1

1

1

1

2

2

1

1

1

1

3

3

4

4

1

1

1

1

1

1

1

1

2

2

1

1

1

1

1

1

2

1

1

1

1

1

1

2

2

1

1

2

2

1

1

1

1

1

1

1

1

1

1

1

1

1

1

1

1

1

1

2

2

1

1

1

1

1

1

1

1

3

3

3

3

2

2

1

1

1

1

10

10

3

3

3

3

1

1

1

1

2

2

2

1

1

3

3

1

1

1

1

1

1

1

1

3

1

1

1

4

4

4

1

1

1

1

1

1

3

2

1

4

4

12

12

1

1

2

2

1

1

2

2

1

1

1

1

1

1

1

1

3

3

4

4

3

3

1

1

3

3

7

7

2

1

1

1

1

1

1

1

1

1

1

2

2

7

3

4

3

3

1

1

1

1

1

1

1

1

1

1

14

14

3

3

1

1

1

1

3

3

1

1

2

2

1

1

1

1

1

1

1

1

1

1

1

1

4

2

1

1

1

1

3

1

1

1

1

1

1

1

5

5

1

1

2

1

1

1

1

1

1

1

1

3

3

1

1

2

1

1

2

2

4

4

1

1

3

3

1

1

3

3

1

1

3

3

8

8

3

3

1

1

1

1

1

1

1

1

6

4

2

2

2

1

1

7

7

2

2

1

1

1

1

556

210

1

1

3

3

1

1

2

2

1

1

40

2

4

7

2

3

1

18

3

1

1

4

2

2

7

7

3

3

1

1

1

1

38

2

2

13

1

16

2

2

3

3

1

1

1

1

2

2

3

3

1

1

1

1

1

1

1

1

2

2

1

1

2

2

33

9

1

1

22

2

2

2

2

1

1

2

2

2

2

1

1

1

1

2

2

1

1

2

2

1

1

2

2

1

1

1

1

1

1

1

1

1

1

2

2

1

1

11

1

1

2

3

1

1

1

1

4

4

1

1

1

1

1

1

2

2

5

5

2

2

1

1

168

1

1

1

1

1

1

1

1

1

1

3

3

1

1

1

1

1

1

1

1

3

3

1

1

1

1

5

5

1

1

3

3

1

1

1

1

2

2

2

1

1

3

3

1

1

1

1

3

3

1

1

1

1

6

6

1

1

1

1

1

1

1

1

2

2

3

3

2

2

2

2

1

1

4

4

1

1

2

2

1

1

4

4

3

3

1

1

1

1

21

3

1

4

5

5

3

1

1

1

1

3

3

1

1

1

1

1

1

3

3

2

2

1

1

1

1

2

1

1

1

1

1

1

1

1

3

1

2

1

1

1

1

1

1

2

2

1

1

1

1

1

1

1

1

1

1

2

2

3

1

2

1

1

5

5

1

1

2

2

1

1

3

3

1

1

3

3

1

1

4

1

3

1

1

1

1

1

1

1

1

1

1

2

2

1

1

5

1

1

1

1

1

1

1

1

1

1

2

1

1

1

1

171

1

1

1

1

1

1

2

2

1

1

1

1

2

2

2

2

1

1

2

2

1

1

1

1

1

1

2

2

1

1

1

1

1

1

1

1

1

1

1

1

2

2

1

1

1

1

1

1

1

1

1

1

1

1

1

1

1

1

3

3

1

1

1

1

1

1

1

1

1

1

1

1

1

1

1

1

1

1

1

1

1

1

2

2

1

1

1

1

1

1

1

1

4

4

1

1

3

3

1

1

1

1

1

1

1

1

1

1

1

1

1

1

1

1

1

1

1

1

1

1

4

4

2

2

1

1

6

6

1

1

1

1

3

3

1

1

1

1

1

1

1

1

1

1

1

1

1

1

1

1

1

1

1

1

1

1

1

1

1

1

1

1

2

2

1

1

1

1

1

1

1

1

1

1

1

1

1

1

2

2

1

1

1

1

1

1

1

1

1

1

1

1

1

1

1

1

1

1

2

2

1

1

1

1

1

1

1

1

1

1

1

1

3

3

1

1

1

1

1

1

1

1

1

1

1

1

1

1

1

1

1

1

1

1

2

2

1

1

1

1

1

1

1

1

1

1

2

2

2

2

1

1

1

1

1

1

1

1

1

1

2

2

1

1

1

1

1

1

2

2

1

1
