# Supplementary material for: Genome and Pangenome Analysis of Lactobacillus hilgardii FLUB—A New Strain Isolated from Mead
Source: Int J Mol Sci. 2021 Apr 6;22(7):3780. doi: 10.3390/ijms22073780 (PMC8038741; doi:10.3390/ijms22073780)
Supplement: Supplementary file 1 [file ijms-22-03780-s001.zip › Supplementary Materials/Interactive charts/Krona COG/Krona_L.hilgardii_COG_complete.html]

Javascript must be enabled to view this page.

magnitude
magnitudeUnassigned

krona

3118

1163

74

1

1

1

1

1

1

2

2

1

1

1

1

1

1

1

1

2

2

1

1

1

1

1

1

1

1

18

18

1

1

2

2

1

1

1

1

1

1

1

1

1

1

2

2

2

2

1

1

5

5

1

1

1

1

5

5

1

1

2

2

1

1

1

1

1

1

1

1

3

3

1

1

2

2

1

1

1

1

1

1

272

2

2

6

6

1

1

1

1

4

4

1

1

1

1

2

2

1

1

1

1

1

1

2

2

2

2

4

4

1

1

1

1

2

2

2

2

1

1

1

1

2

2

2

2

4

3

1

1

1

1

1

1

1

3

3

1

1

8

8

5

5

1

1

5

5

2

2

2

2

1

1

2

2

6

6

1

1

2

1

1

2

2

3

3

1

1

3

3

1

1

1

1

1

1

1

1

1

1

2

2

64

64

1

1

1

1

1

1

1

1

1

1

2

2

5

5

1

1

1

1

1

1

1

1

4

4

1

1

2

2

1

1

3

3

2

2

1

1

4

4

1

1

1

1

1

1

3

3

1

1

2

2

1

1

1

1

1

1

1

1

2

2

2

2

5

5

1

1

1

1

1

1

24

24

3

3

1

1

1

1

1

1

3

3

2

2

1

1

1

1

1

1

3

3

2

2

3

3

289

1

1

1

1

1

1

1

1

1

1

1

1

1

1

1

1

1

1

1

1

1

1

1

1

1

1

1

1

1

1

3

3

1

1

1

1

6

6

1

1

6

6

9

9

6

6

1

1

1

1

5

5

1

1

9

9

1

1

3

3

12

12

1

1

2

2

2

2

1

1

2

2

1

1

6

6

1

1

1

1

1

1

17

15

2

1

1

1

1

1

1

1

1

1

1

3

3

1

1

1

1

1

1

5

5

1

1

1

1

8

8

1

1

1

1

1

1

1

1

4

4

2

2

1

1

1

1

1

1

1

1

3

3

1

1

1

1

1

1

2

2

3

3

1

1

1

1

1

1

2

2

1

1

2

2

2

2

4

4

1

1

1

1

2

2

1

1

1

1

2

2

1

1

1

1

2

2

2

2

1

1

1

1

1

1

3

3

1

1

3

3

4

4

1

1

3

3

4

4

1

1

1

1

1

1

3

3

1

1

2

2

2

2

1

1

1

1

2

2

1

1

6

6

1

1

7

7

1

1

1

1

2

2

1

1

2

2

2

2

4

4

3

3

2

2

1

1

1

1

3

3

4

4

1

1

1

1

2

2

2

2

2

2

1

1

1

1

2

1

1

2

2

1

1

133

1

1

2

2

2

2

4

4

1

1

2

2

1

1

1

1

8

8

1

1

1

1

2

2

1

1

1

1

3

3

2

2

3

3

2

2

1

1

2

2

1

1

5

5

1

1

7

7

1

1

1

1

1

1

1

1

1

1

4

4

2

2

1

1

1

1

1

1

4

4

1

1

3

3

1

1

2

2

1

1

2

2

1

1

1

1

1

1

1

1

1

1

1

1

2

2

1

1

2

2

2

2

1

1

2

2

1

1

2

2

1

1

1

1

3

3

1

1

1

1

4

4

1

1

1

1

1

1

1

1

1

1

9

9

1

1

1

1

1

1

1

1

2

2

162

2

2

2

2

1

1

1

1

2

2

1

1

1

1

1

1

2

2

1

1

3

3

5

5

2

2

2

2

2

2

3

3

3

3

2

2

1

1

2

2

2

2

1

1

3

3

1

1

1

1

3

3

1

1

3

3

1

1

1

1

1

1

1

1

1

1

1

1

5

5

1

1

3

3

2

2

3

3

1

1

2

2

2

2

1

1

2

2

1

1

6

6

3

3

1

1

3

3

4

4

3

3

1

1

1

1

1

1

1

1

1

1

1

1

4

4

1

1

4

4

1

1

2

2

1

1

1

1

2

2

18

8

10

4

3

1

1

1

1

1

3

3

2

2

1

1

5

2

3

1

1

1

1

27

1

1

1

1

1

1

1

1

1

1

1

1

1

1

1

1

3

3

1

1

1

1

3

3

1

1

1

1

1

1

1

1

1

1

3

3

1

1

1

1

1

1

102

1

1

3

3

1

1

1

1

2

2

1

1

1

1

2

2

2

2

1

1

1

1

1

1

1

1

1

1

1

1

1

1

1

1

2

2

1

1

2

2

1

1

1

1

1

1

1

1

1

1

2

2

1

1

4

4

1

1

1

1

2

2

3

3

1

1

2

2

1

1

2

2

1

1

1

1

1

1

1

1

1

1

1

1

1

1

2

2

1

1

1

1

1

1

1

1

1

1

2

2

1

1

4

4

1

1

2

2

3

3

1

1

2

2

2

2

3

3

1

1

4

4

2

2

1

1

2

2

2

2

2

2

1

1

104

2

2

1

1

1

1

1

1

1

1

1

1

1

1

1

1

1

1

1

1

1

1

2

2

1

1

1

1

1

1

1

1

3

3

1

1

1

1

1

1

2

2

4

4

2

2

4

4

1

1

1

1

1

1

1

1

1

1

1

1

1

1

2

2

1

1

1

1

1

1

1

1

1

1

1

1

2

2

1

1

8

8

1

1

1

1

1

1

1

1

1

1

2

2

1

1

1

1

4

4

1

1

5

5

1

1

1

1

1

1

1

1

1

1

1

1

2

2

2

2

1

1

1

1

1

1

7

7

1

1

1

1

1

1

612

2

1

1

1

1

219

2

2

1

1

1

1

4

2

2

1

1

7

7

1

1

41

7

4

3

1

18

2

4

2

1

1

1

1

1

1

2

2

5

5

1

1

2

2

1

1

2

2

2

2

3

3

1

1

2

2

1

1

1

1

2

2

33

22

1

9

1

3

3

1

1

1

1

2

2

1

1

2

2

2

2

1

1

40

2

13

3

2

2

16

2

3

3

1

1

1

1

1

1

13

2

1

3

1

3

1

1

1

2

2

2

2

4

4

2

2

1

1

1

1

2

2

1

1

1

1

1

1

1

1

1

1

2

2

1

1

1

1

1

1

4

4

177

1

1

2

2

1

1

6

6

4

4

1

1

2

2

1

1

1

1

1

1

1

1

1

1

1

1

1

1

1

1

1

1

1

1

2

2

1

1

1

1

1

1

1

1

1

1

2

2

1

1

1

1

1

1

1

1

1

1

1

1

2

2

1

1

1

1

1

1

1

1

1

1

1

1

1

1

1

1

1

1

1

1

1

1

1

1

1

1

3

3

1

1

1

1

1

1

1

1

1

1

1

1

1

1

1

1

1

1

1

1

1

1

1

1

1

1

1

1

1

1

1

1

1

1

1

1

1

1

1

1

1

1

2

2

2

2

1

1

1

1

1

1

3

3

1

1

1

1

1

1

1

1

1

1

1

1

3

3

1

1

3

3

2

2

1

1

1

1

1

1

1

1

1

1

1

1

1

1

1

1

1

1

2

2

1

1

2

2

1

1

1

1

3

3

1

1

1

1

1

1

1

1

1

1

1

1

1

1

1

1

1

1

1

1

1

1

1

1

1

1

1

1

1

1

1

1

1

1

3

3

1

1

3

3

1

1

1

1

1

1

2

2

1

1

1

1

1

1

1

1

1

1

1

1

1

1

1

1

2

2

1

1

4

4

1

1

1

1

1

1

2

2

1

1

1

1

1

1

209

1

1

1

1

1

1

1

1

1

1

2

1

1

1

1

1

1

3

3

2

2

1

1

1

1

5

5

1

1

1

1

5

5

1

1

2

2

2

2

4

4

3

2

1

2

2

1

1

3

3

1

1

2

2

1

1

1

1

1

1

1

1

1

1

2

2

2

2

1

1

1

1

1

1

1

1

1

1

1

1

1

1

1

1

2

2

1

1

1

1

1

1

3

3

3

3

1

1

41

11

1

7

5

3

4

10

1

1

1

1

3

3

5

5

1

1

3

3

1

1

3

1

2

1

1

1

1

1

1

1

1

1

1

1

1

2

2

1

1

6

6

1

1

1

1

1

1

1

1

3

3

2

2

1

1

2

2

1

1

5

5

2

1

1

3

3

6

6

1

1

3

3

4

4

2

2

1

1

1

1

1

1

3

3

1

1

3

3

2

2

4

3

1

2

2

5

1

1

1

1

1

1

1

1

1

1

790

368

1

1

5

5

1

1

2

2

3

2

1

4

4

1

1

1

1

1

1

8

8

1

1

1

1

2

2

1

1

3

1

1

1

1

1

1

1

1

1

1

1

3

3

1

1

1

1

1

1

1

1

1

1

1

1

2

2

1

1

1

1

1

1

1

1

1

1

1

1

8

8

1

1

1

1

4

4

3

3

1

1

1

1

4

4

3

1

2

1

1

1

1

1

1

3

3

1

1

1

1

1

1

3

3

2

2

2

1

1

1

1

1

1

3

3

1

1

1

1

4

4

2

2

1

1

1

1

1

1

1

1

3

3

3

3

1

1

2

2

2

2

1

1

1

1

1

1

4

4

4

4

1

1

3

3

1

1

1

1

1

1

3

3

2

2

1

1

1

1

1

1

2

2

1

1

3

3

1

1

4

4

2

2

1

1

1

1

1

1

1

1

1

1

4

1

1

1

1

1

1

1

1

1

1

4

4

2

2

1

1

3

1

1

1

3

3

3

3

1

1

1

1

3

3

1

1

1

1

1

1

1

1

1

1

1

1

1

1

1

1

2

2

1

1

1

1

2

1

1

1

1

1

1

3

3

1

1

1

1

4

4

7

3

4

1

1

7

7

1

1

12

12

1

1

1

1

1

1

1

1

1

1

1

1

1

1

3

3

2

2

1

1

1

1

1

1

2

2

1

1

1

1

2

1

1

2

2

2

2

6

4

2

5

5

1

1

1

1

1

1

2

2

2

2

1

1

4

4

4

1

1

2

1

1

3

3

1

1

2

2

1

1

2

2

3

3

1

1

2

1

1

2

1

1

1

1

6

6

10

10

1

1

1

1

1

1

7

7

1

1

4

4

14

14

1

1

422

1

1

2

1

1

3

3

1

1

14

1

1

5

1

2

1

1

1

1

1

1

2

1

1

1

1

2

2

1

1

2

2

1

1

2

2

5

1

2

2

1

1

86

1

4

1

1

1

1

4

1

2

1

3

2

1

3

2

5

2

1

1

2

4

1

1

1

1

2

1

2

2

3

1

1

1

1

1

6

1

1

5

1

1

2

1

6

2

2

10

10

1

1

1

1

1

1

1

1

1

1

1

1

109

2

2

1

3

1

1

1

1

4

1

1

1

2

1

1

1

1

1

1

1

1

1

1

1

1

1

1

1

2

1

1

1

1

3

1

1

1

1

1

6

1

1

1

1

4

1

2

2

1

2

1

2

1

2

1

1

1

1

2

1

1

1

1

1

1

1

1

1

1

1

1

1

1

1

1

1

1

1

1

1

1

1

1

1

2

2

2

2

1

1

6

6

1

1

8

1

1

1

1

1

1

1

1

1

1

1

1

2

2

1

1

144

1

1

1

1

1

1

1

1

1

1

1

1

1

1

1

1

1

1

1

5

1

2

1

1

1

1

1

1

1

1

1

3

2

2

1

1

1

1

1

1

1

1

1

1

1

1

2

5

1

2

1

2

1

1

1

1

1

1

1

1

1

1

1

1

2

1

2

1

1

2

1

2

1

4

1

4

1

1

2

1

1

1

1

1

1

1

1

1

1

1

1

1

1

1

1

1

1

1

1

1

1

1

2

1

7

1

2

2

1

1

553

26

1

1

2

2

1

1

1

1

1

1

3

3

1

1

2

2

1

1

1

1

4

4

1

1

1

1

1

1

2

2

1

1

1

1

1

1

92

2

2

1

1

2

2

1

1

1

1

1

1

11

11

2

2

1

1

1

1

1

1

1

1

4

4

1

1

1

1

2

2

2

2

8

8

1

1

2

2

2

2

1

1

1

1

15

11

2

2

5

5

2

2

5

5

2

2

4

4

3

3

1

1

1

1

4

4

57

2

2

1

1

1

1

2

2

1

1

1

1

1

1

2

2

4

4

1

1

1

1

1

1

7

7

1

1

1

1

1

1

1

1

1

1

15

15

2

2

2

2

1

1

2

2

1

1

3

2

1

1

1

72

1

1

1

1

9

9

1

1

1

1

2

2

5

5

7

7

4

4

1

1

2

2

15

15

3

3

1

1

1

1

3

1

2

7

7

1

1

2

2

3

3

1

1

1

1

31

1

1

2

2

1

1

1

1

1

1

1

1

1

1

1

1

2

2

1

1

1

1

2

2

1

1

1

1

1

1

1

1

2

2

3

3

1

1

1

1

1

1

2

1

1

1

1

1

1

4

1

1

1

1

2

2

175

5

5

6

6

2

2

2

2

2

2

2

2

1

1

3

3

1

1

1

1

2

2

1

1

1

1

1

1

2

2

1

1

11

9

2

1

1

2

2

1

1

4

4

2

2

7

7

2

1

1

1

1

1

1

2

2

3

3

2

2

1

1

1

1

1

1

1

1

2

2

2

2

1

1

1

1

3

3

1

1

1

1

2

2

1

1

1

1

1

1

1

1

1

1

8

8

2

2

4

4

5

2

3

1

1

1

1

1

1

1

1

1

1

1

1

1

1

3

3

1

1

2

2

2

2

2

2

3

3

1

1

2

2

4

4

2

2

2

2

2

2

1

1

1

1

4

4

1

1

1

1

1

1

1

1

1

1

1

1

3

3

6

6

4

4

1

1

3

3

96

1

1

2

2

2

2

1

1

1

1

1

1

1

1

1

1

1

1

2

2

1

1

1

1

3

3

1

1

3

1

2

2

2

1

1

2

1

1

1

1

1

1

1

1

1

1

2

2

1

1

1

1

1

1

1

1

2

2

1

1

1

1

1

1

2

2

1

1

10

10

1

1

1

1

4

2

2

2

2

1

1

2

2

4

3

1

1

1

1

1

2

2

1

1

1

1

1

1

2

2

1

1

4

4

1

1

2

2

1

1

1

1

1

1

1

1

2

2

1

1

1

1

1

1
