# Supplementary material for: Genome and Pangenome Analysis of Lactobacillus hilgardii FLUB—A New Strain Isolated from Mead
Source: Int J Mol Sci. 2021 Apr 6;22(7):3780. doi: 10.3390/ijms22073780 (PMC8038741; doi:10.3390/ijms22073780)
Supplement: Supplementary file 1 [file ijms-22-03780-s001.zip › Supplementary Materials/Interactive charts/Krona COG/Krona_L.hilgardii_COG_plasmid4.html]

Javascript must be enabled to view this page.

magnitude
magnitudeUnassigned

krona

6

1

1

1

1

4

3

1

1

1

1

1

1

1

1

1

1

1

1

1
