# Supplementary material for: Genome and Pangenome Analysis of Lactobacillus hilgardii FLUB—A New Strain Isolated from Mead
Source: Int J Mol Sci. 2021 Apr 6;22(7):3780. doi: 10.3390/ijms22073780 (PMC8038741; doi:10.3390/ijms22073780)
Supplement: Supplementary file 1 [file ijms-22-03780-s001.zip › Supplementary Materials/Interactive charts/Krona COG/Krona_pangenome_COG_accesory.html]

Javascript must be enabled to view this page.

magnitude
magnitudeUnassigned

krona

1210

430

15

1

1

1

1

1

1

2

2

1

1

2

2

1

1

1

1

1

1

1

1

1

1

2

2

10

1

1

2

2

1

1

1

1

1

1

1

1

1

1

1

1

1

1

132

2

2

14

14

1

1

2

2

1

1

4

4

1

1

1

1

2

2

1

1

2

2

2

2

2

2

1

1

4

4

5

5

1

1

2

2

1

1

3

3

7

7

16

16

6

6

2

2

1

1

2

1

1

1

1

1

1

1

1

2

2

2

2

1

1

4

4

2

2

1

1

3

3

1

1

1

1

1

1

2

2

1

1

1

1

1

1

2

2

1

1

1

1

2

2

1

1

1

1

1

1

3

3

1

1

1

1

2

2

2

2

1

1

1

1

48

1

1

1

1

2

2

1

1

1

1

2

2

2

2

1

1

1

1

1

1

1

1

1

1

1

1

1

1

1

1

3

3

1

1

1

1

1

1

1

1

1

1

1

1

1

1

1

1

1

1

1

1

1

1

1

1

2

2

1

1

2

2

1

1

1

1

2

2

2

2

1

1

1

1

1

1

1

1

71

1

1

1

1

3

3

1

1

1

1

2

2

1

1

1

1

2

2

2

2

1

1

1

1

1

1

1

1

1

1

1

1

1

1

1

1

3

3

1

1

4

4

1

1

1

1

1

1

2

2

1

1

2

2

1

1

2

2

2

2

2

2

1

1

2

2

2

2

1

1

1

1

1

1

1

1

1

1

1

1

2

2

1

1

1

1

1

1

1

1

1

1

2

2

1

1

1

1

3

2

1

19

1

1

6

6

1

1

4

4

1

1

1

1

1

1

1

1

3

3

93

2

2

1

1

3

3

1

1

1

1

1

1

1

1

2

2

1

1

1

1

1

1

1

1

1

1

1

1

1

1

1

1

1

1

1

1

2

2

3

3

1

1

1

1

1

1

1

1

2

2

1

1

1

1

1

1

3

3

2

2

1

1

1

1

2

2

6

6

5

5

1

1

1

1

1

1

1

1

1

1

1

1

1

1

1

1

1

1

1

1

1

1

2

2

1

1

1

1

1

1

2

2

2

1

1

1

1

3

1

2

2

2

1

1

2

2

1

1

2

2

1

1

1

1

2

2

1

1

42

1

1

1

1

2

2

1

1

1

1

1

1

1

1

2

2

1

1

1

1

1

1

2

2

1

1

1

1

1

1

2

2

1

1

1

1

1

1

1

1

2

2

3

3

4

4

1

1

1

1

1

1

1

1

2

2

1

1

2

2

235

36

1

1

2

2

3

3

2

2

4

4

1

1

1

1

2

2

7

7

1

1

1

1

9

9

1

1

1

1

64

2

2

1

1

1

1

2

2

1

1

1

1

1

1

1

1

1

1

1

1

1

1

1

1

4

4

2

2

2

2

2

2

1

1

1

1

2

2

1

1

1

1

2

2

1

1

1

1

2

2

2

2

4

4

1

1

2

2

1

1

1

1

4

2

2

1

1

1

1

2

2

1

1

1

1

2

2

2

2

2

2

1

1

10

1

1

1

1

1

1

2

2

1

1

1

1

1

1

2

2

33

1

1

1

1

1

1

1

1

2

2

1

1

1

1

1

1

3

3

1

1

1

1

1

1

1

1

1

1

1

1

2

2

1

1

3

3

4

4

4

4

1

1

13

1

1

1

1

2

2

3

3

1

1

1

1

1

1

1

1

1

1

1

1

33

2

1

1

1

1

1

1

2

2

1

1

1

1

1

1

1

1

1

1

1

1

2

2

1

1

1

1

2

2

1

1

1

1

1

1

1

1

3

3

1

1

1

1

1

1

2

1

1

2

2

1

1

42

1

1

1

1

1

1

1

1

1

1

1

1

1

1

2

2

1

1

1

1

1

1

28

28

2

2

4

1

1

2

2

1

1

327

149

1

1

3

3

1

1

3

1

1

1

1

1

1

1

1

1

1

1

1

1

1

1

1

1

1

1

1

1

1

1

1

1

1

1

1

1

3

3

1

1

1

1

1

1

1

1

1

1

1

1

1

1

1

1

1

1

2

2

1

1

1

1

1

1

1

1

1

1

3

3

1

1

2

2

3

1

2

2

2

1

1

6

6

1

1

1

1

2

1

1

1

1

2

2

1

1

1

1

2

2

1

1

1

1

1

1

2

2

1

1

1

1

1

1

2

1

1

1

1

1

1

3

3

1

1

1

1

1

1

2

2

1

1

1

1

2

2

3

3

1

1

1

1

3

3

2

2

1

1

1

1

2

2

1

1

2

2

1

1

1

1

1

1

1

1

1

1

4

1

1

1

1

1

1

3

3

3

3

1

1

3

3

2

2

2

2

1

1

1

1

1

1

3

1

2

1

1

2

2

1

1

2

2

2

1

1

1

1

178

8

2

2

2

1

1

1

1

2

2

2

2

1

1

1

1

1

1

1

1

43

1

1

1

2

1

2

1

1

1

1

1

5

1

1

3

1

1

1

2

1

1

1

1

1

1

1

1

1

2

1

2

1

1

1

1

1

1

1

3

1

2

60

1

1

1

1

1

1

1

2

2

1

2

2

1

1

1

1

1

1

1

1

1

1

1

1

1

1

2

1

1

3

1

2

1

3

2

1

1

1

1

1

2

1

1

1

1

1

1

1

1

1

29

2

2

1

2

3

1

1

1

1

2

2

2

1

2

1

2

1

1

1

1

1

1

1

1

1

1

1

3

2

1

1

1

4

1

1

1

1

8

8

1

1

1

1

218

3

1

1

1

1

1

1

108

1

1

1

1

1

1

1

1

2

2

7

7

1

1

1

1

2

2

12

1

1

1

1

2

1

1

4

2

2

16

10

5

1

2

2

1

1

2

2

1

1

1

1

18

2

8

1

1

1

5

3

3

1

1

1

1

1

1

23

17

2

4

1

1

1

1

1

1

1

1

1

1

2

2

81

1

1

1

1

3

3

1

1

5

5

2

2

1

1

1

1

1

1

1

1

2

2

2

2

1

1

1

1

1

1

1

1

1

1

1

1

3

3

2

2

2

1

1

1

1

1

1

1

1

1

1

1

1

1

1

1

1

2

2

1

1

1

1

1

1

1

1

1

1

25

2

4

8

1

1

3

3

3

1

1

2

2

1

1

1

1

1

1

1

1

1

1

22

1

1

2

2

1

1

1

1

1

1

1

1

1

1

1

1

2

2

1

1

1

1

1

1

1

1

1

1

1

1

1

1

1

1

1

1

1

1

1

1

4

2

2

1

1

1

1
