# Supplementary material for: Genome and Pangenome Analysis of Lactobacillus hilgardii FLUB—A New Strain Isolated from Mead
Source: Int J Mol Sci. 2021 Apr 6;22(7):3780. doi: 10.3390/ijms22073780 (PMC8038741; doi:10.3390/ijms22073780)
Supplement: Supplementary file 1 [file ijms-22-03780-s001.zip › Supplementary Materials/Interactive charts/Krona COG/Krona_pangenome_COG_core.html]

Javascript must be enabled to view this page.

magnitude
magnitudeUnassigned

krona

2059

358

118

1

1

1

1

1

1

2

2

1

1

1

1

1

1

1

1

2

1

1

1

1

4

1

3

1

1

1

1

4

4

6

6

4

4

4

4

1

1

1

1

3

3

1

1

3

3

1

1

3

3

5

5

1

1

1

1

1

1

1

1

1

1

1

1

1

1

1

1

1

1

2

2

1

1

1

1

1

1

2

2

1

1

2

2

1

1

1

1

1

1

5

5

1

1

3

3

1

1

2

2

4

2

2

2

2

1

1

1

1

3

3

1

1

1

1

3

3

2

2

2

2

1

1

2

2

2

2

1

1

3

3

1

1

28

1

1

1

1

1

1

1

1

1

1

1

1

2

2

2

2

1

1

4

4

1

1

1

1

2

2

1

1

2

1

1

1

1

1

1

1

1

2

2

1

1

1

1

1

70

1

1

1

1

1

1

1

1

2

2

1

1

1

1

1

1

1

1

1

1

1

1

1

1

4

2

2

1

1

2

2

1

1

1

1

2

1

1

1

1

1

1

1

1

1

1

2

2

7

7

1

1

1

1

1

1

2

2

1

1

1

1

1

1

1

1

1

1

1

1

1

1

1

1

1

1

2

2

1

1

1

1

1

1

4

4

2

2

2

2

1

1

1

1

1

1

1

1

2

2

48

1

1

1

1

1

1

3

3

2

2

1

1

3

3

1

1

1

1

2

1

1

2

2

3

3

4

4

1

1

9

9

10

10

1

1

1

1

1

1

22

1

1

1

1

3

3

1

1

1

1

1

1

1

1

2

1

1

2

2

1

1

1

1

1

1

1

1

1

1

1

1

1

1

2

2

58

1

1

7

7

2

2

1

1

1

1

3

3

1

1

1

1

1

1

1

1

1

1

1

1

1

1

1

1

1

1

2

2

1

1

1

1

8

8

2

2

1

1

12

2

8

2

2

2

1

1

4

4

13

1

1

1

1

1

1

1

1

1

1

3

3

1

1

2

2

1

1

1

1

503

251

1

1

2

2

1

1

1

1

7

7

3

3

2

2

1

1

3

3

2

2

3

1

1

1

3

3

1

1

1

1

2

2

2

2

2

2

1

1

1

1

1

1

1

1

1

1

2

2

2

2

1

1

3

3

1

1

2

1

1

2

2

4

1

3

6

6

1

1

1

1

6

6

1

1

1

1

1

1

6

6

3

3

1

1

1

1

1

1

1

1

1

1

1

1

1

1

3

3

4

4

1

1

2

2

1

1

1

1

2

2

1

1

1

1

4

1

1

2

2

2

4

4

1

1

1

1

1

1

2

2

1

1

1

1

1

1

1

1

2

2

2

2

9

9

1

1

2

2

3

3

4

4

1

1

4

4

1

1

1

1

1

1

1

1

5

3

2

1

1

2

2

3

3

3

3

2

2

1

1

1

1

1

1

1

1

1

1

1

1

1

1

1

1

2

2

1

1

1

1

1

1

1

1

1

1

1

1

1

1

3

3

1

1

2

2

1

1

1

1

1

1

3

3

1

1

1

1

12

12

1

1

1

1

3

3

1

1

1

1

1

1

1

1

2

2

1

1

1

1

3

2

1

4

4

1

1

1

1

10

10

1

1

252

1

1

1

1

4

2

2

53

1

1

1

1

1

4

1

1

1

1

1

1

2

2

1

1

1

1

1

1

1

1

4

5

1

1

2

1

2

1

1

1

1

1

4

1

2

2

67

2

1

1

1

1

3

1

2

1

1

1

1

1

1

1

1

1

1

1

1

1

1

1

2

1

1

1

1

1

1

2

4

1

2

1

1

1

1

1

1

1

2

1

1

1

1

1

1

1

1

1

1

1

1

1

1

1

1

1

1

1

1

9

1

1

1

3

1

1

1

6

6

2

2

86

1

1

1

1

1

1

1

1

1

1

1

1

1

1

1

1

1

1

1

1

1

2

1

1

1

2

1

1

1

1

1

3

4

1

1

1

1

1

1

1

1

1

1

1

1

1

1

3

1

1

1

1

1

1

1

1

1

2

1

1

1

4

1

5

1

1

1

1

1

1

1

1

1

1

1

1

1

1

1

2

2

1

1

2

2

2

2

5

1

1

1

1

1

1

1

389

154

1

1

1

1

1

1

2

2

1

1

1

1

1

1

2

2

1

1

1

1

1

1

2

2

1

1

1

1

1

1

2

2

1

1

1

1

2

2

1

1

1

1

1

1

1

1

1

1

1

1

1

1

1

1

2

2

6

6

1

1

1

1

1

1

1

1

1

1

2

2

1

1

1

1

1

1

1

1

1

1

1

1

1

1

1

1

1

1

1

1

1

1

1

1

1

1

1

1

1

1

1

1

1

1

1

1

1

1

1

1

1

1

1

1

4

4

1

1

1

1

1

1

1

1

1

1

1

1

1

1

1

1

1

1

1

1

1

1

1

1

1

1

1

1

1

1

1

1

1

1

1

1

1

1

2

2

2

2

1

1

1

1

1

1

1

1

1

1

1

1

1

1

1

1

1

1

2

2

1

1

1

1

1

1

2

2

1

1

1

1

1

1

1

1

1

1

1

1

1

1

2

2

1

1

1

1

1

1

1

1

1

1

1

1

1

1

1

1

1

1

1

1

3

3

1

1

1

1

1

1

1

1

1

1

1

1

1

1

1

1

2

2

1

1

1

1

1

1

1

1

1

1

1

1

1

1

1

1

1

1

1

1

125

3

3

26

1

9

11

1

1

1

2

1

1

1

1

3

3

2

2

1

1

4

2

2

23

2

4

1

3

2

2

2

7

1

1

2

2

1

1

1

1

1

1

1

1

3

3

2

2

1

1

1

1

1

1

1

1

1

1

1

1

1

1

1

1

1

1

1

1

1

1

19

1

13

5

1

1

1

1

4

1

1

1

1

1

1

1

1

2

2

1

1

2

2

3

3

1

1

1

1

1

1

1

1

1

3

1

1

1

1

1

1

106

2

2

1

1

2

1

1

5

5

1

1

3

3

1

1

4

4

1

1

1

1

1

1

2

2

1

1

3

3

1

1

1

1

3

2

1

1

1

1

1

1

1

1

1

3

2

1

2

2

1

1

2

2

2

2

1

1

2

2

1

1

1

1

1

1

1

1

1

1

1

1

1

1

1

1

1

1

2

2

1

1

1

1

1

1

2

2

1

1

1

1

1

1

1

1

1

1

2

2

1

1

1

1

1

1

1

1

2

2

1

1

1

1

1

1

1

1

1

1

3

3

2

2

1

1

2

2

1

1

1

1

1

1

1

1

1

1

1

1

1

1

1

1

1

1

1

1

1

1

1

1

1

1

1

1

809

225

1

1

5

5

2

2

1

1

1

1

1

1

3

3

1

1

1

1

1

1

1

1

1

1

1

1

1

1

5

5

1

1

1

1

1

1

1

1

1

1

1

1

1

1

5

5

8

8

1

1

5

5

1

1

1

1

1

1

1

1

1

1

9

9

2

2

1

1

1

1

2

2

2

2

1

1

1

1

3

3

1

1

1

1

1

1

1

1

1

1

1

1

4

4

1

1

3

3

1

1

2

2

1

1

1

1

1

1

1

1

2

2

3

3

1

1

1

1

6

6

2

2

1

1

1

1

4

4

1

1

5

5

2

2

1

1

1

1

1

1

1

1

1

1

1

1

1

1

1

1

2

2

1

1

7

7

3

3

1

1

1

1

1

1

2

2

1

1

1

1

6

6

1

1

1

1

1

1

2

2

4

4

1

1

1

1

1

1

2

2

12

10

2

1

1

1

1

1

1

2

2

1

1

1

1

1

1

1

1

6

6

3

3

2

2

1

1

2

1

1

1

1

1

1

1

1

1

1

1

1

1

1

1

1

1

1

2

2

1

1

1

1

1

1

15

1

1

1

1

1

1

1

1

1

1

1

1

1

1

2

2

1

1

1

1

1

1

1

1

1

1

1

1

85

1

1

1

1

1

1

1

1

1

1

1

1

1

1

1

1

2

2

2

2

1

1

2

2

1

1

1

1

1

1

1

1

2

2

1

1

1

1

2

2

1

1

2

2

1

1

1

1

1

1

1

1

1

1

2

2

2

2

1

1

1

1

4

4

1

1

1

1

2

2

1

1

1

1

1

1

2

2

1

1

1

1

1

1

3

3

1

1

1

1

1

1

1

1

1

1

2

2

2

2

2

2

1

1

1

1

2

2

1

1

2

2

3

3

3

3

1

1

1

1

1

1

94

1

1

1

1

1

1

1

1

3

3

1

1

1

1

1

1

1

1

1

1

1

1

1

1

3

3

1

1

1

1

2

2

3

3

1

1

1

1

5

5

1

1

1

1

1

1

1

1

1

1

1

1

8

8

2

2

1

1

1

1

4

4

1

1

2

2

2

2

1

1

2

2

1

1

1

1

1

1

1

1

2

2

6

6

1

1

2

2

1

1

1

1

1

1

1

1

1

1

7

7

1

1

1

1

1

1

1

1

2

2

60

1

1

1

1

1

1

1

1

3

3

1

1

1

1

1

1

1

1

1

1

1

1

1

1

1

1

2

2

1

1

15

15

1

1

1

1

1

1

1

1

1

1

1

1

1

1

1

1

1

1

1

1

2

2

2

2

2

2

2

2

3

3

1

1

1

1

1

1

2

2

1

1

98

1

1

1

1

2

2

2

2

4

4

5

5

1

1

1

1

1

1

1

1

2

2

2

2

2

2

2

2

2

2

2

2

1

1

2

2

2

2

1

1

1

1

2

2

1

1

1

1

6

6

2

2

2

2

2

2

1

1

2

2

2

2

1

1

14

8

6

1

1

1

1

2

2

1

1

1

1

4

4

1

1

3

3

3

2

1

1

1

1

1

4

2

2

1

1

69

1

1

1

1

1

1

1

1

1

1

2

2

1

1

2

2

1

1

1

1

1

1

4

4

1

1

2

2

2

2

4

4

1

1

1

1

1

1

1

1

2

2

1

1

1

1

1

1

1

1

2

2

4

4

4

4

1

1

1

1

1

1

1

1

1

1

2

2

6

6

1

1

1

1

1

1

1

1

2

2

1

1

1

1

1

1

1

1

163

1

1

1

1

1

1

1

1

1

1

1

1

2

2

1

1

1

1

1

1

2

2

2

2

8

8

1

1

1

1

1

1

1

1

1

1

2

2

1

1

2

2

1

1

1

1

1

1

1

1

2

2

1

1

1

1

2

2

1

1

1

1

1

1

3

3

1

1

7

7

1

1

1

1

2

2

2

2

1

1

3

3

1

1

2

2

1

1

1

1

1

1

1

1

1

1

51

51

1

1

3

3

1

1

1

1

2

2

1

1

3

3

1

1

1

1

1

1

2

2

1

1

2

2

4

4

1

1

2

2

2

2

1

1

2

1

1

1

1

1

1

2

2

1

1
