# Supplementary material for: Genome and Pangenome Analysis of Lactobacillus hilgardii FLUB—A New Strain Isolated from Mead
Source: Int J Mol Sci. 2021 Apr 6;22(7):3780. doi: 10.3390/ijms22073780 (PMC8038741; doi:10.3390/ijms22073780)
Supplement: Supplementary file 1 [file ijms-22-03780-s001.zip › Supplementary Materials/Interactive charts/Krona COG/Krona_pangenome_COG_singletons.html]

Javascript must be enabled to view this page.

magnitude
magnitudeUnassigned

krona

905

297

215

2

2

1

1

1

1

1

1

4

4

1

1

2

2

1

1

2

2

1

1

1

1

1

1

1

1

5

5

1

1

1

1

108

1

16

33

14

1

18

12

7

6

1

1

2

2

1

1

1

1

2

2

1

1

1

1

1

1

2

2

1

1

5

5

1

1

2

2

1

1

1

1

1

1

2

2

37

37

5

5

1

1

1

1

1

1

5

5

1

1

1

1

1

1

1

1

1

1

3

1

1

1

1

1

1

22

1

1

1

1

1

1

1

1

1

1

1

1

1

1

2

2

1

1

3

3

1

1

1

1

2

2

1

1

1

1

1

1

1

1

1

1

57

1

1

19

2

2

4

1

9

1

1

1

1

1

1

1

1

1

1

1

1

1

1

1

2

2

1

1

2

1

1

1

1

1

1

1

1

1

1

1

1

3

1

2

1

1

1

1

2

2

1

1

6

1

2

2

1

2

2

1

1

1

1

2

2

230

49

1

1

1

1

1

1

1

1

1

1

1

1

2

2

1

1

1

1

2

2

1

1

1

1

1

1

1

1

1

1

2

2

1

1

2

1

1

1

1

1

1

1

1

1

1

3

3

1

1

1

1

1

1

1

1

1

1

1

1

1

1

1

1

1

1

2

2

1

1

2

2

1

1

3

3

1

1

1

1

9

1

1

3

3

1

1

1

1

1

1

1

1

1

1

14

1

1

1

1

1

1

1

1

3

3

1

1

1

1

1

1

1

1

1

1

1

1

1

1

36

1

1

1

1

1

1

1

1

1

1

1

1

1

1

1

1

1

1

1

1

1

1

1

1

6

1

5

1

1

1

1

1

1

1

1

1

1

3

3

2

2

3

3

1

1

1

1

1

1

1

1

1

1

29

2

2

1

1

1

1

1

1

1

1

1

1

1

1

1

1

1

1

1

1

1

1

1

1

1

1

1

1

1

1

2

2

3

3

2

2

2

1

1

1

1

1

1

1

1

1

1

12

5

5

1

1

1

1

1

1

1

1

1

1

2

2

59

1

1

2

2

1

1

1

1

1

1

1

1

2

2

1

1

1

1

1

1

1

1

1

1

2

2

8

8

2

2

3

3

1

1

1

1

1

1

17

17

1

1

1

1

1

1

2

2

2

2

2

1

1

1

1

22

1

1

2

2

1

1

1

1

2

2

1

1

1

1

1

1

2

2

1

1

2

2

1

1

1

1

1

1

1

1

1

1

1

1

1

1

166

13

1

1

1

1

1

1

1

1

1

1

4

4

2

2

1

1

1

1

14

1

1

1

1

1

1

2

2

1

1

1

1

2

2

1

1

1

1

1

1

1

1

1

1

28

6

6

1

1

2

2

7

7

2

2

1

1

1

1

1

1

1

1

4

4

2

1

1

25

1

1

1

1

2

2

1

1

1

1

2

2

1

1

1

1

2

2

4

4

1

1

6

3

1

1

1

1

1

1

1

26

3

3

4

1

3

2

2

5

5

1

1

1

1

3

3

1

1

1

1

2

2

1

1

1

1

1

1

1

1

1

8

1

1

1

1

2

2

1

1

1

1

1

1

1

1

51

1

1

1

1

8

8

1

1

1

1

2

2

5

5

1

1

1

1

1

1

1

1

2

2

4

4

7

7

1

1

1

1

1

1

1

1

1

1

2

2

1

1

1

1

2

2

1

1

1

1

1

1

1

1

212

129

2

1

1

1

1

1

1

31

1

1

1

1

1

1

1

1

1

1

1

1

1

1

1

1

1

1

2

1

1

1

1

1

1

1

2

1

1

1

1

4

4

1

1

4

1

1

1

1

1

1

44

1

1

1

1

1

2

1

2

1

1

2

1

1

2

1

2

1

1

1

1

1

1

1

1

2

1

1

1

1

1

1

1

1

2

3

3

1

1

1

2

2

3

3

1

1

1

1

2

2

23

1

1

1

1

1

1

1

2

1

3

2

2

1

1

2

1

1

3

3

1

1

83

2

2

1

1

1

1

1

1

1

1

1

1

1

1

2

2

1

1

1

1

1

1

1

1

1

1

1

1

1

1

1

1

1

1

1

1

1

1

1

1

1

1

1

1

1

1

2

2

1

1

1

1

1

1

3

3

1

1

1

1

1

1

1

1

1

1

1

1

1

1

3

3

1

1

2

2

2

2

1

1

1

1

2

2

1

1

1

1

1

1

1

1

1

1

1

1

1

1

3

3

1

1

1

1

4

4

1

1

1

1

1

1

2

2

1

1

2

2

1

1

1

1

1

1

1

1

1

1

1

1

1

1
