# Supplementary material for: Genome and Pangenome Analysis of Lactobacillus hilgardii FLUB—A New Strain Isolated from Mead
Source: Int J Mol Sci. 2021 Apr 6;22(7):3780. doi: 10.3390/ijms22073780 (PMC8038741; doi:10.3390/ijms22073780)
Supplement: Supplementary file 1 [file ijms-22-03780-s001.zip › Supplementary Materials/Supplementary Materials/AMR genes/Plasmid_replication_proteins_MSA.pdf]

Aligned using an external algorithm

Consensus

1. fig|1588.38.peg.1
2. fig|1588.36.peg.6
3. fig|1588.37.peg.7
4. fig|1578.1144.peg.1
5. fig|1588.35.peg.1

```
  -------MTKXTXFNXYXADNVYGALFFQFPKVLMYXXQYXXLSNDAKLAYMVLKDRLEYSLRNXWVDEXXHXYFIFTNQ
1 MSEIFRDKTKNGKVRPWRERKIENVRYAEYLSILEFKRAHDIRGCGETLRFRKINNQLK--LYQTW----------FCQK  68
2 -------MNQD---NFYKVSEVYAETYYQLPKVLFTNDNYKGLSNDAKVAFALLKDRFNYSVRNHWVDEQDNLYFVYTNQ  70
3 --------MDQTNFKFYQADNVYGALFFQFPKVLMYSQQYRGLSDAAKLAYMVLKDRLEYSLRNHWIDEEGHVYFIFTNQ  72
4 -------MTKSTDFNYYEADNVYGALFFQFPKVLMYGEQYRHLSNDAKLAYMVLKDRLEYSLRNNWVDEDNHIYFIFTNQ  73
5 -------MTKSTNFNYYEADNVYGALFFQFPKVLMYGEQYKHLSNDAKLAYMVLKDRLEYSLRNNWVDSKGHVYFIFTNQ  73
```

```
  ELXXLFNCSXXKXIKIKXXLEXIGLLXQKXXGFNPKTXKNEPNRLYLXKLDVXATDVYLRGEYXQXXXQXLXTSXXXKXX
1 RLCPL--CNWRKSMKNSSQLKQI---------IAEAVSRQPKGRFLFLTLTVK--NAY-GAEELRSSLRVLTKAFNKLTR  134
2 ALKEILNCSEPKIIKIKKNLETAHLLYQKRIGI------NQPNRLYLLKPEVTANDVY----------------------  122
3 ELKDLFDCSNDKLAAVKKDLERAGLLYQKAMHFNPKTGKNEPNRLYLAELDMQSTDVYLRGEYAQKEPQTLATSENPKIG  152
4 ELGNLFNCSEHKVIKIKAELESIGLLFQKQLGFNPKTKKNEPNRLYLSKLDVQATDVYLRGEYGQKDAQSLGTSGTAKNA  153
5 ELMDLFNCSKGKVIKIKAELESIGLLVQKQMGFNPKTKKNEPNRLYLSKLDVKATDVYLRGEYGQSSPQTLATSGSSKNE  153
```

```
  XXXXTXXXLXXSGXXKXXV--PXKXVDXXXQTLXTSGXXKXXXNLXKEXKXRDKXRYNIDTQKLDFSTAXFS--------
1 YKKVTKNLLGYLRSTEITVNDEDRSYNQHLHVLLFVKSSYFTGNNTNYIKQAEWTKLWQRALKVDYEPVVHVEIVKANKR  214
2 -RQVSSQSLDNSGTKNSLV--PVNSTNTDCQTLDNSGTKNSLVNLYKEHKDIDKSRYNIDTQKLDFSTAQFS--------  191
3 RSRETAQTLATSENPKIGR--SRKFVDDTPQTLATSENPKIGHDLDKELKERDTNRYNIDTQKLDFSTAQFS--------  222
4 VPHKTAESLGISGTAKNAV--PHKTVDKDAQSLGTSGTAKNAVNLDLERSDRDKDRYLIDTPELDFSTANYS--------  223
5 LPRETVETLATSGSSKNEL--PHKFVDNSSQTLATSGSSKNGLNLYKEPKERDKKRYNIDTQKLDFSTAHFS--------  223
```

```
  ------PAEXEKQNKDLVNHANDFLTDEDSGLPVFLEPEAVQLLSFWCRTPQQMRRFIGIILNAKYRVEKDHQDIGVXIP
1 KGTGSLQASAEETAKYEVKSSDYMTADDDRNLEVINDLE-------YALAGTRQISYGGLFKQIKQDLKLEDVDKGDLVH  287
2 ------PAELEKQNKDLVNHANDFLTDEDSGLPVFLEPEAVQLLSFWCRTPKQMRRFIGIILNAKYRVEKDHQDIGVIIP  265
3 ------PAELEKQNKDLVNHANDFLTDEDSGLPVFLEPEAVQLLSFWCRTPQQMRRFIGIILNAKYRVEKDHQDIGVLIP  296
4 ------QAEIEQQNRDLIENAYHFLTHSETN-DIFLEPEAVQLISFWARTPQQMRRFIKIILNAKYKVEKEHQDIGVYII  296
5 ------PAEIQKQNQDLVNHANDFLTDEDSGLPVFLEPEAVQLLSFWCRTPQQMRRFIGIILNAKYRVEKDHKDIGVIIP  297
```

```
  LDDEELKPLMTKALRRYFNALRSNEKHIKNVENYLYGTMQNLFGXWWNKXAXXXYXAKHPEX----XXDQXXXXX
1 VGNEEY-----------------SKEQMEAAEEVVAK---------WD------------------FKKQNYFMW  318
2 LDDKELKPLMTKALRRYFNALRSNEKHIKNIENYLYGTMQNLFGIWWNKRANKQWKAKHKPK----FTDQ-----  331
3 LDDEELKSLMTKALRRYFNALRSNEKHIKNVENYLYGTMQNLFGVWWNKKANAKYQAEHPET----GQHQAALFN  367
4 LDDPELKPLMTQTLRRYFNVLRSNEKHVKNVENYLYGTMQNLFGIWWNKQAAREYAAKHPEEQ---NTDNEPYWN  368
5 LDDEELKPLMTKALRRYFNALRSNEKHIKNVENYLYGTMQNLFGVWWNKQAAREYAAKHPEEEKSADNDNSGLYY  372
```

Consensus Threshold: >50%

Compare to: the consensus

Amino acids that match the reference are marked with yellow highlighting.

Created: czwartek, 25 mar 2021

Last Modified: czwartek, 25 mar 2021
