# Supplementary material for: Genome and Pangenome Analysis of Lactobacillus hilgardii FLUB—A New Strain Isolated from Mead
Source: Int J Mol Sci. 2021 Apr 6;22(7):3780. doi: 10.3390/ijms22073780 (PMC8038741; doi:10.3390/ijms22073780)
Supplement: Supplementary file 1 [file ijms-22-03780-s001.zip › Supplementary Materials/Supplementary Materials/Rplots.pdf]

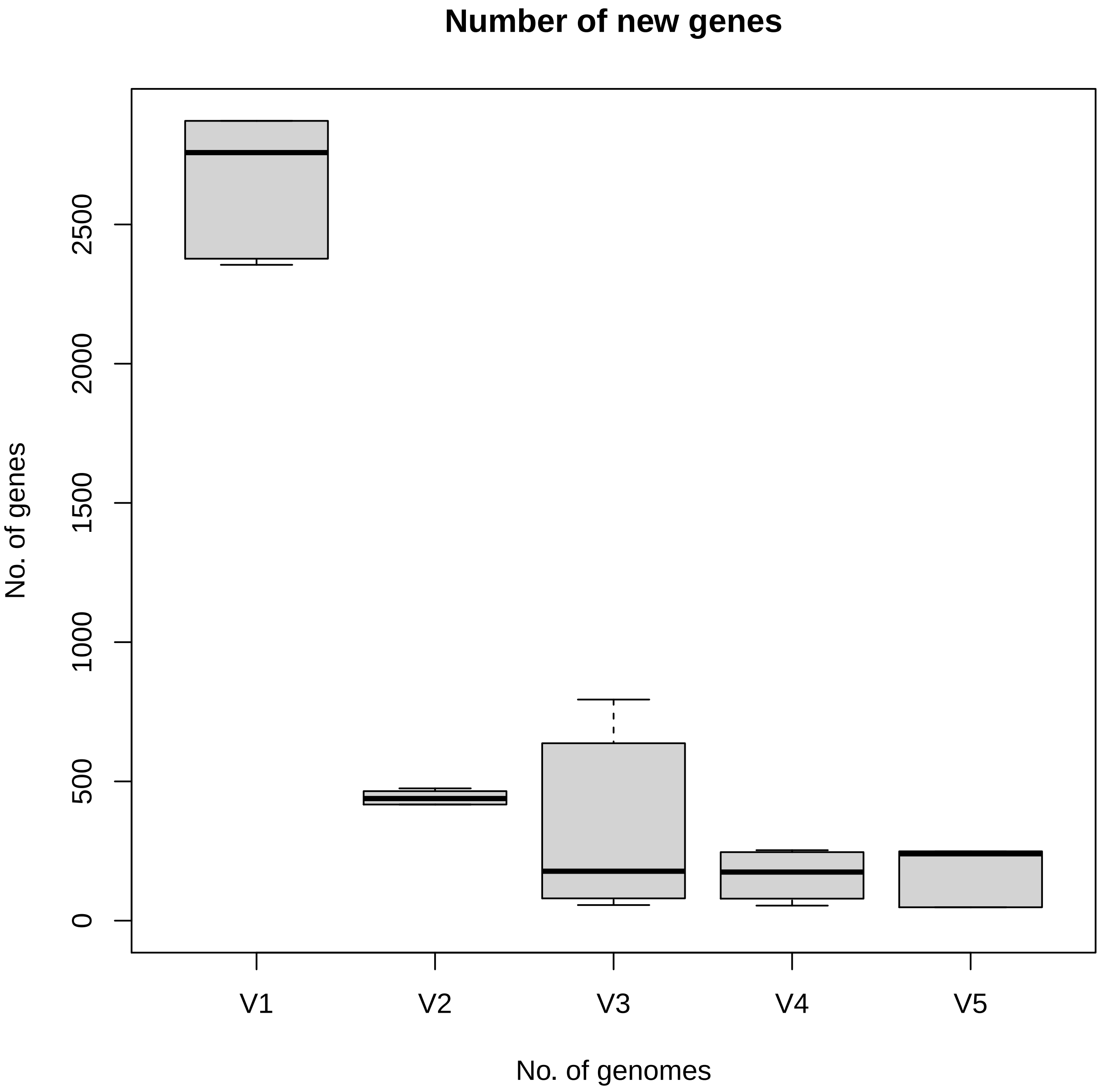

Number of new genes
No. of genes
0
500
1000
1500
2000
2500
V1
V2
V3
V4
V5
No. of genomes

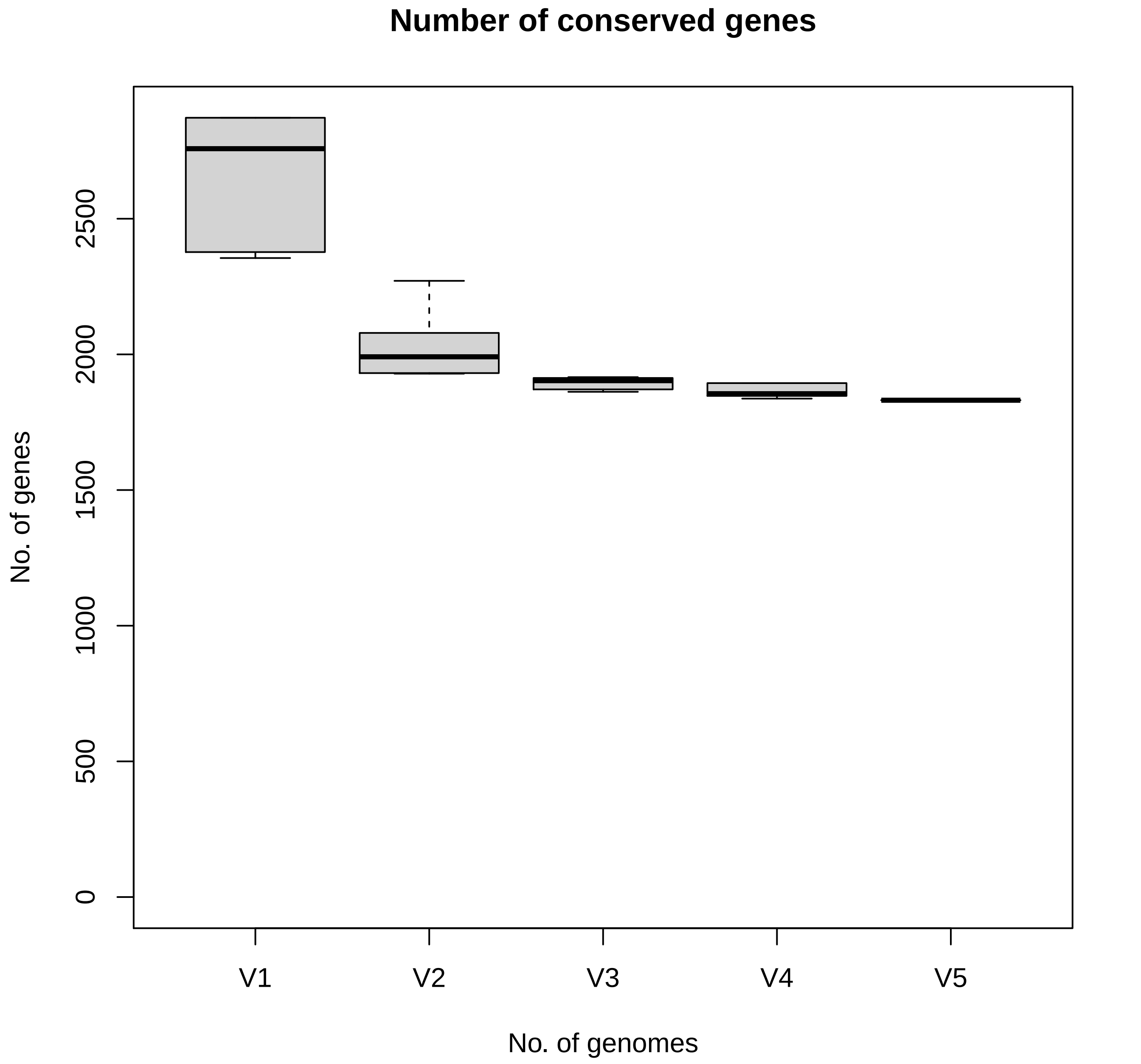

Number of conserved genes
No. of genes
0
500
1000
1500
2000
2500
V1
V2
V3
V4
V5
No. of genomes

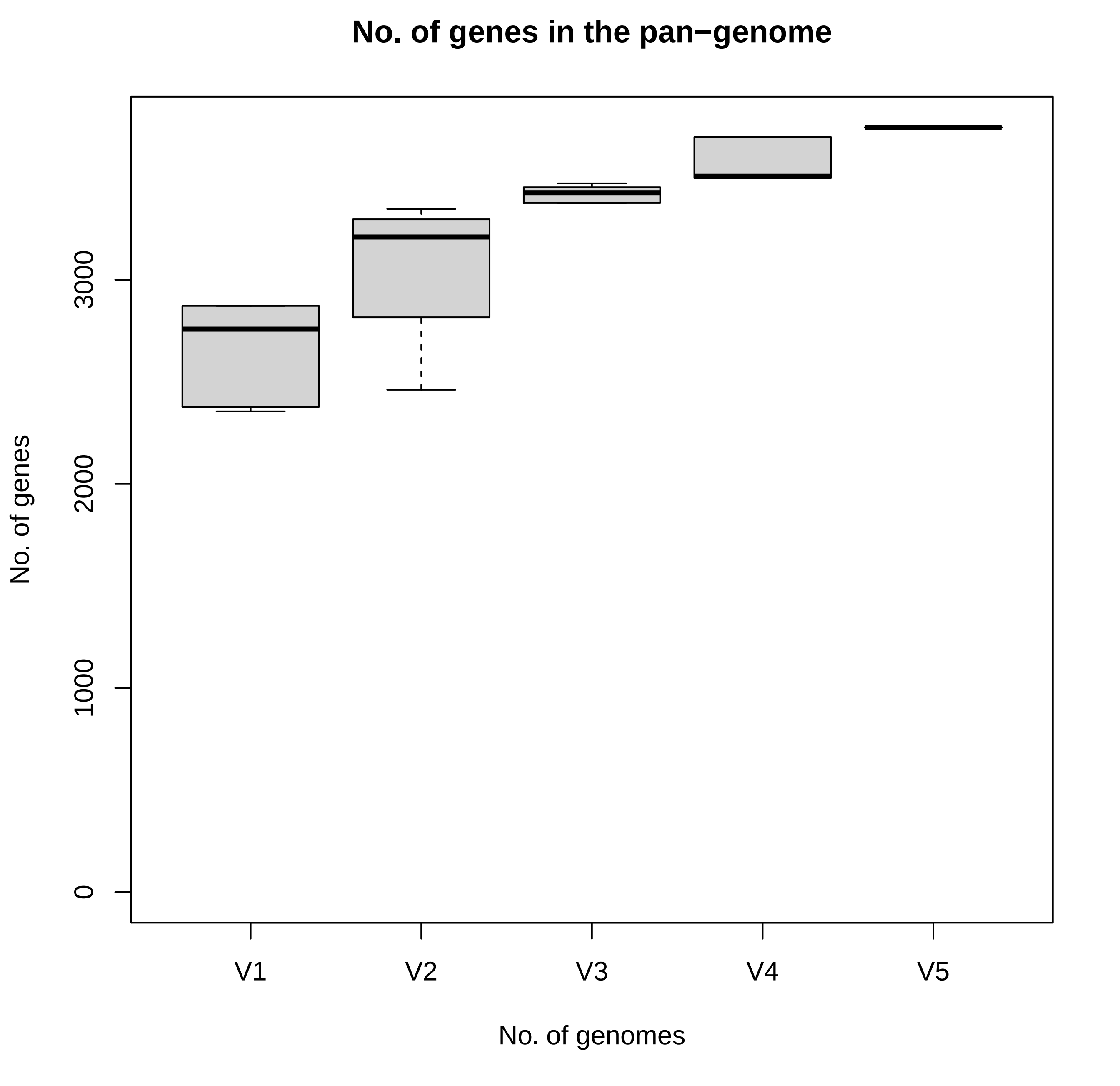
No. of genes in the pan−genome
No. of genes
0
1000
2000
3000
V1
V2
V3
V4
V5
No. of genomes

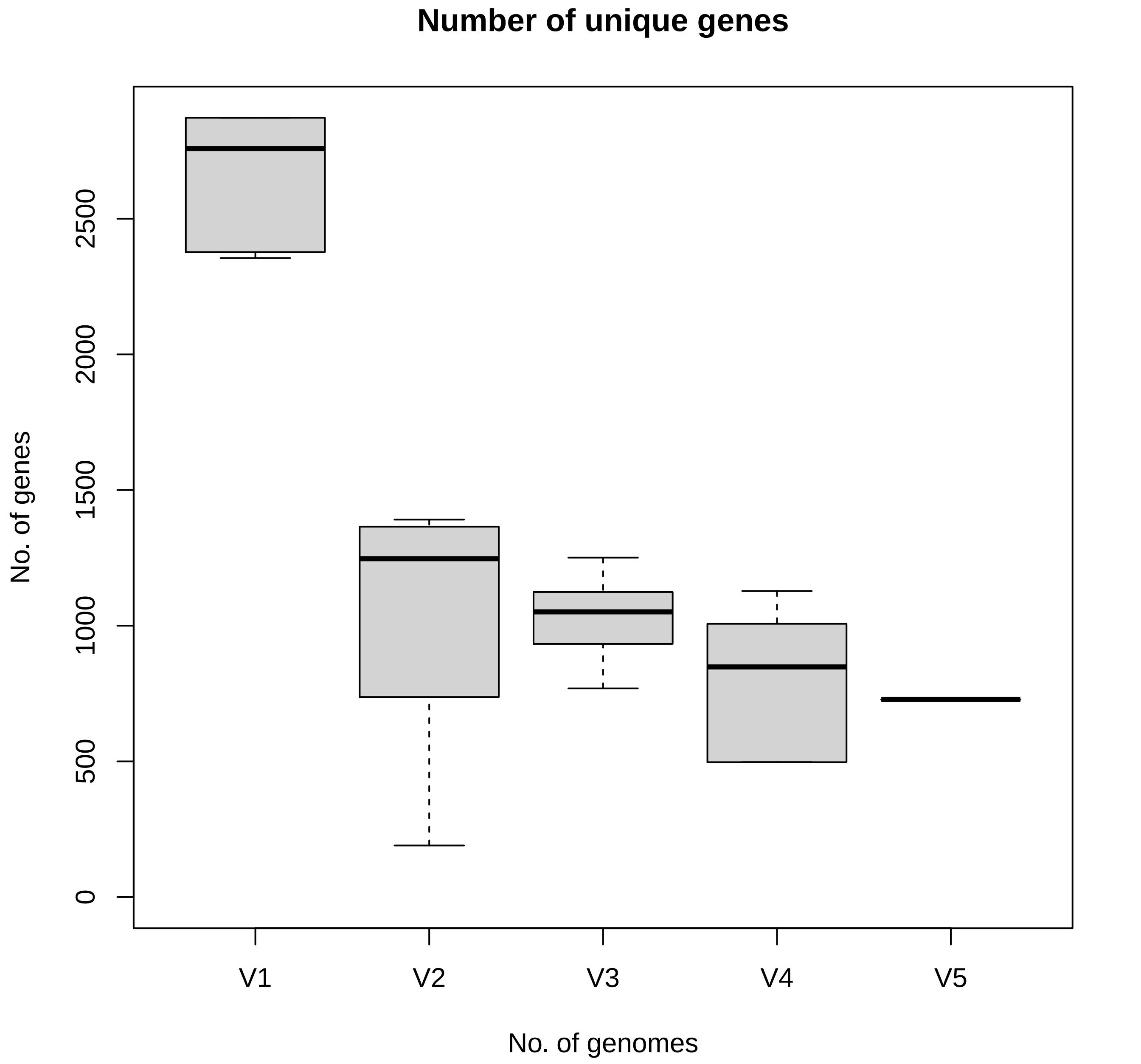

Number of unique genes
No. of genes
0
500
1000
1500
2000
2500
V1
V2
V3
V4
V5
No. of genomes

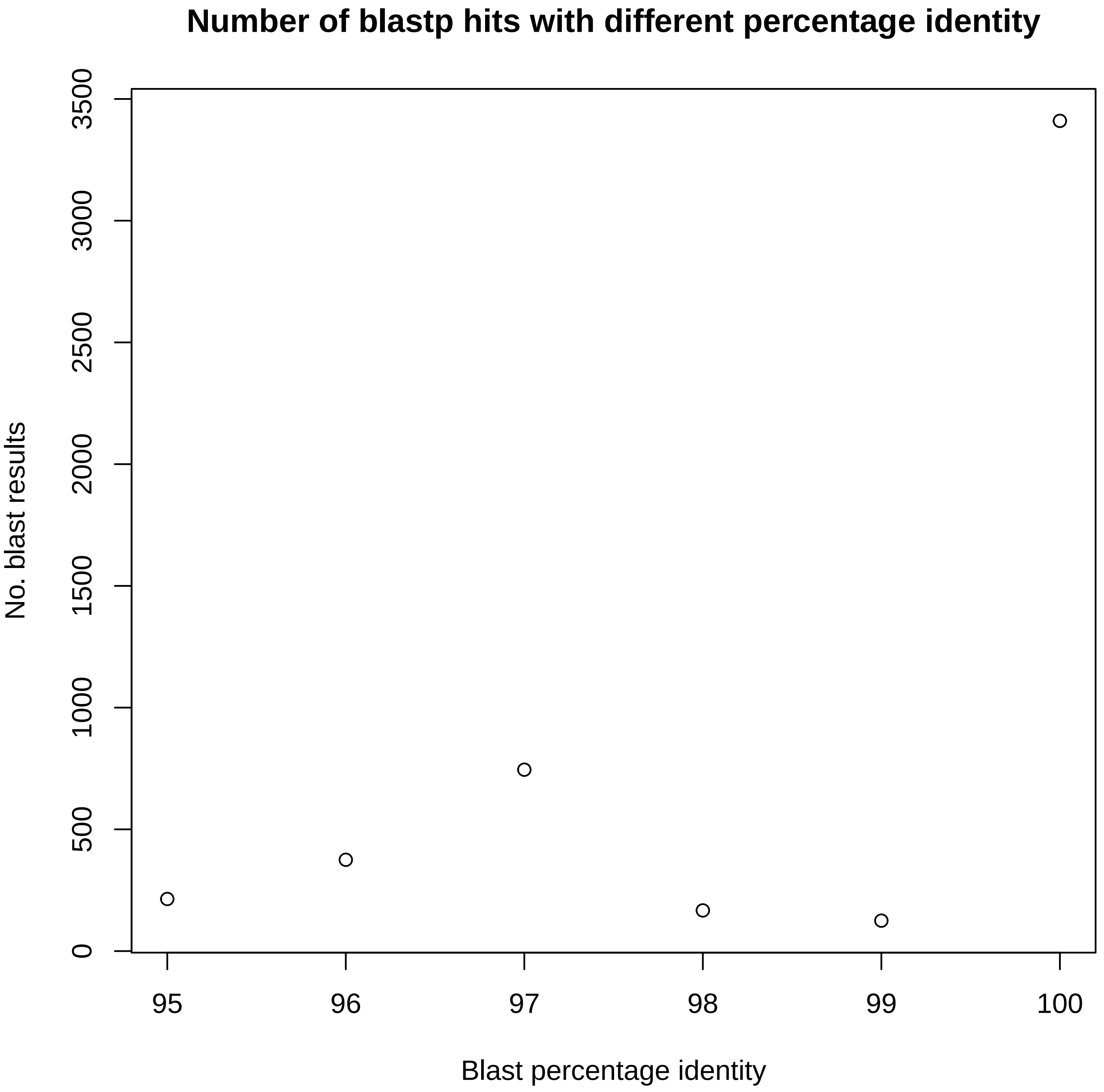

Number of blastp hits with different percentage identity
No. blast results
0
500
1000
1500
2000
2500
3000
3500
95
96
97
98
99
100
Blast percentage identity

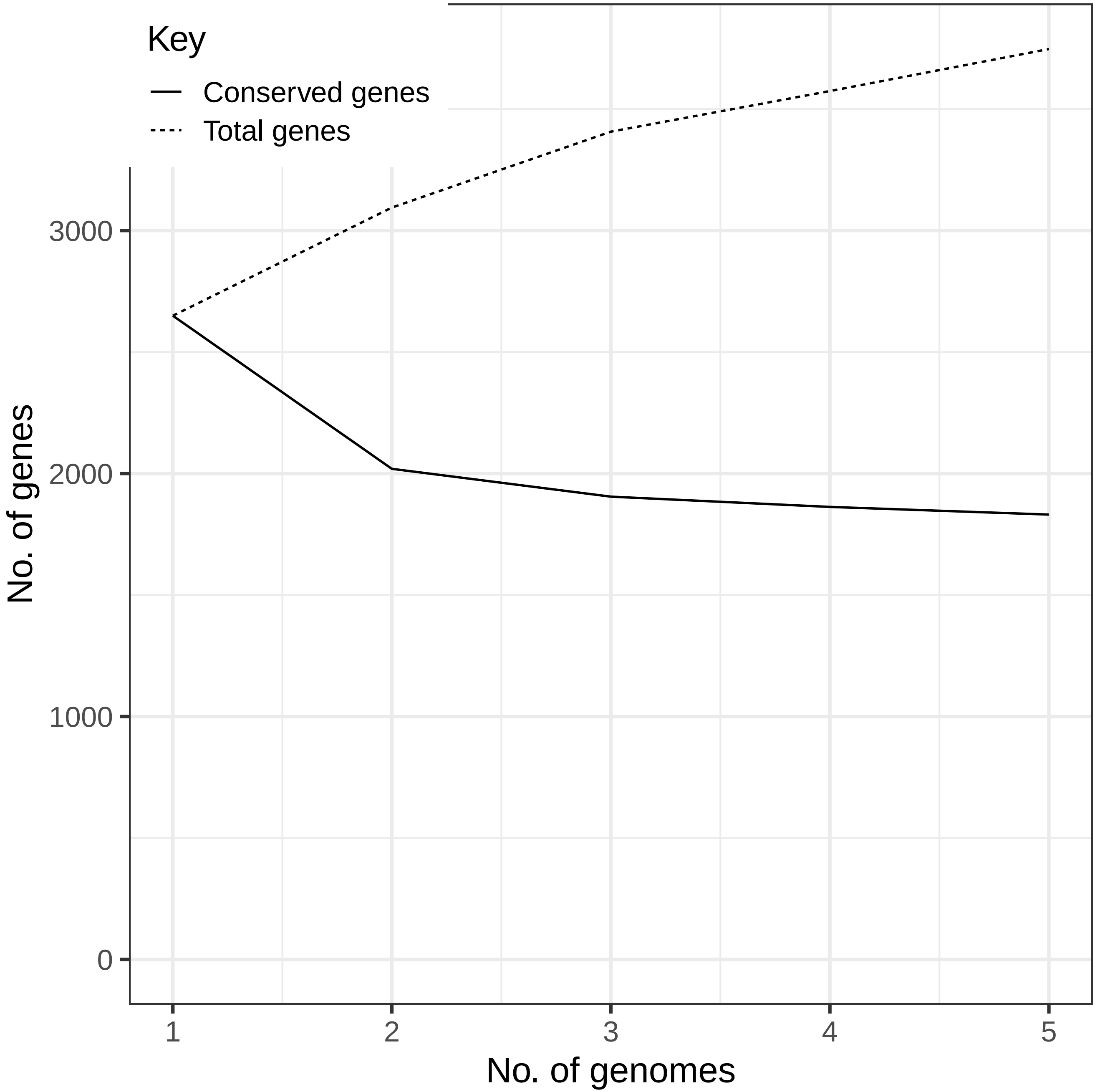
Key
Conserved genes
Total genes
No. of genes
3000
2000
1000
0
1
2
3
4
5
No. of genomes

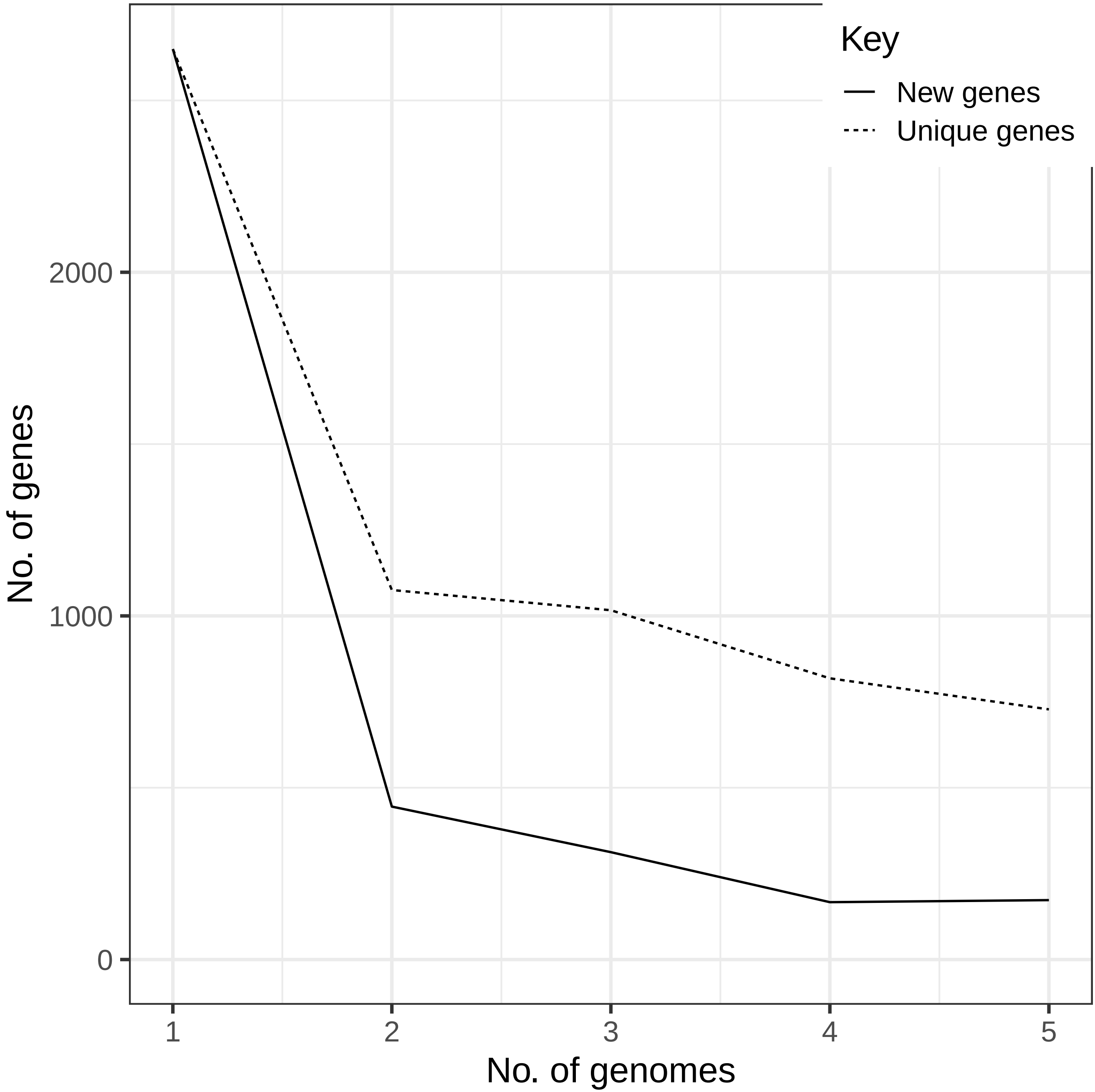

Key
New genes
Unique genes
No. of genes
2000
1000
0
1
2
3
4
5
No. of genomes
