# Supplementary figures and images for: Genome and Pangenome Analysis of Lactobacillus hilgardii FLUB—A New Strain Isolated from Mead
Source: Int J Mol Sci. 2021 Apr 6;22(7):3780. doi: 10.3390/ijms22073780 (PMC8038741; doi:10.3390/ijms22073780)

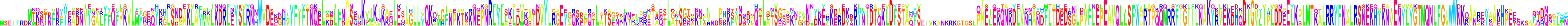

Supplement: Supplementary file 1 [file ijms-22-03780-s001.zip › Supplementary Materials/Supplementary Materials/AMR genes/Plasmid_replication_proteins_MSA_all.png]

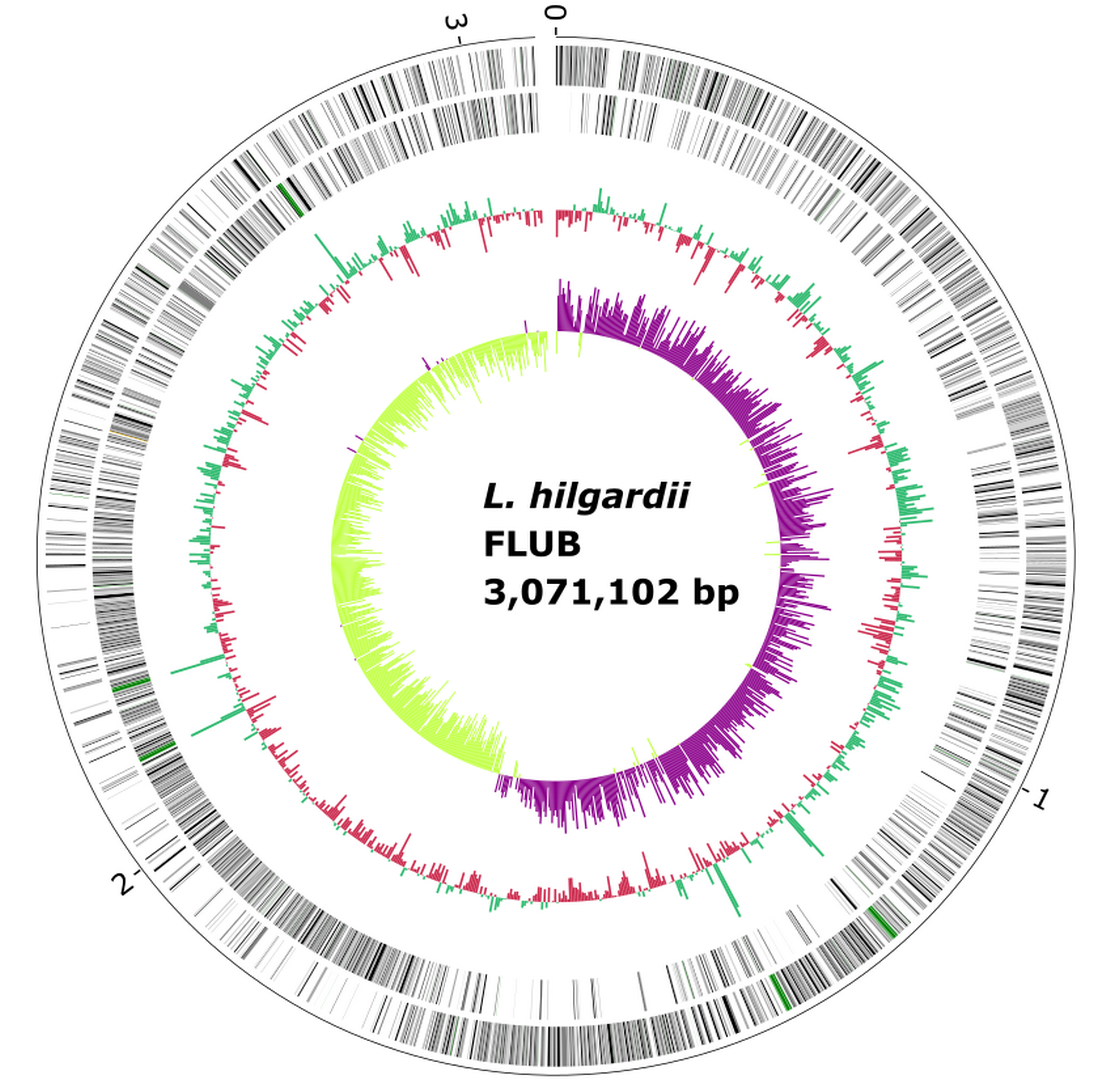

Supplement: Supplementary file 1 [file ijms-22-03780-s001.zip › Supplementary Materials/Supplementary Materials/CGView/CGView_chromosome_L.hilgardii.tif]

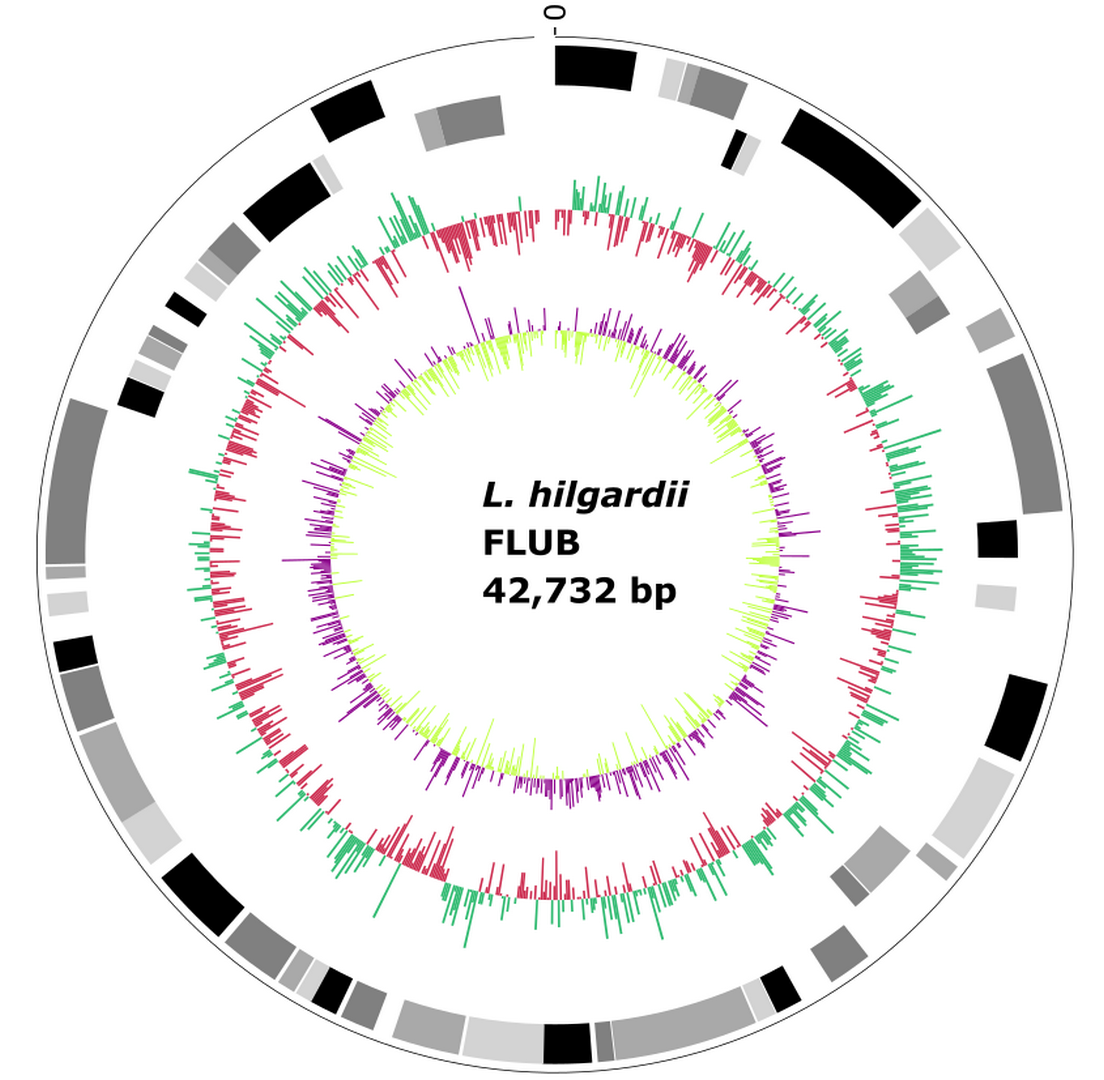

Supplement: Supplementary file 1 [file ijms-22-03780-s001.zip › Supplementary Materials/Supplementary Materials/CGView/CGView_plasmid1_L.hilgardii.tif]

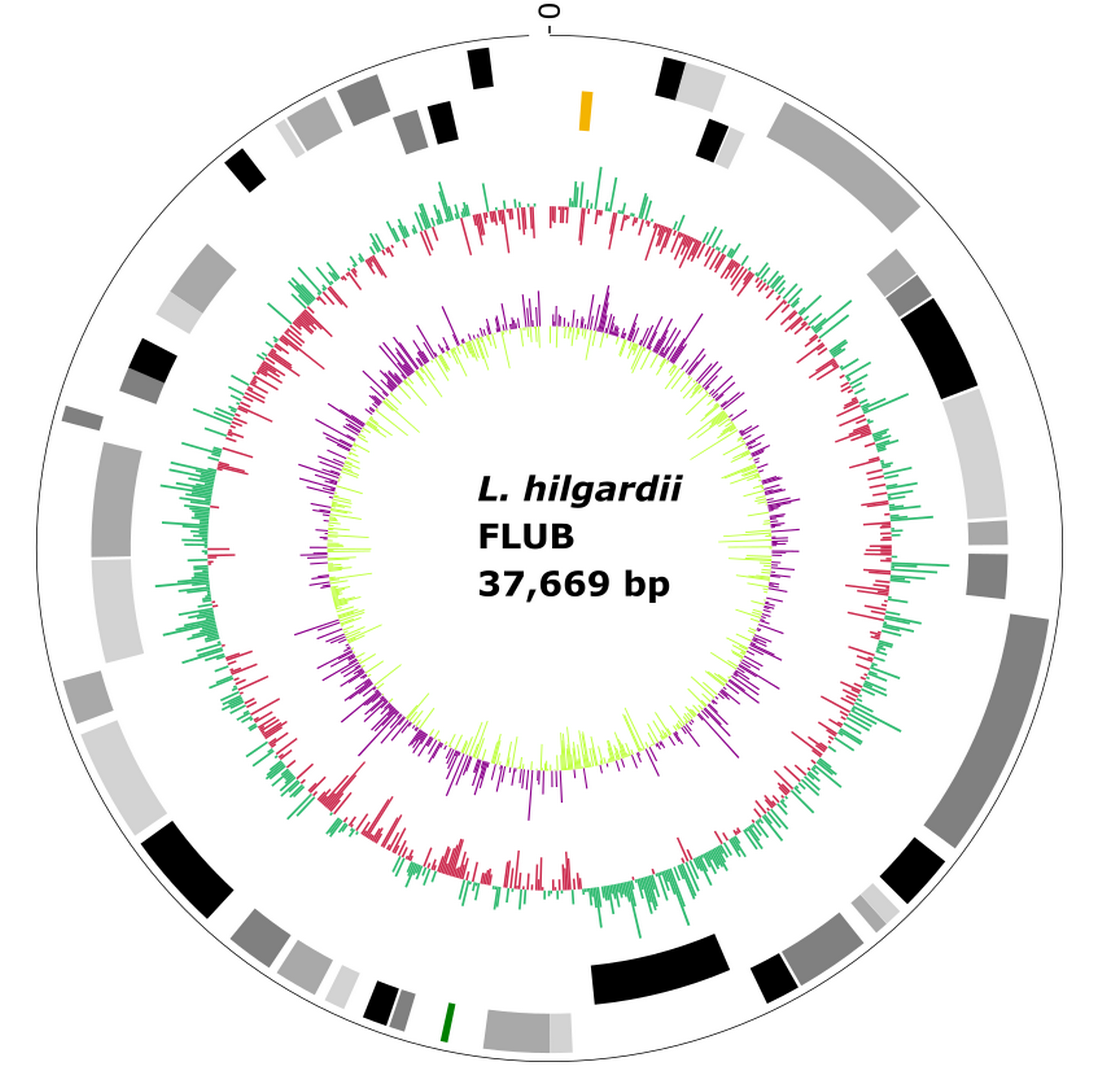

Supplement: Supplementary file 1 [file ijms-22-03780-s001.zip › Supplementary Materials/Supplementary Materials/CGView/CGView_plasmid2_L.hilgardii.tif]

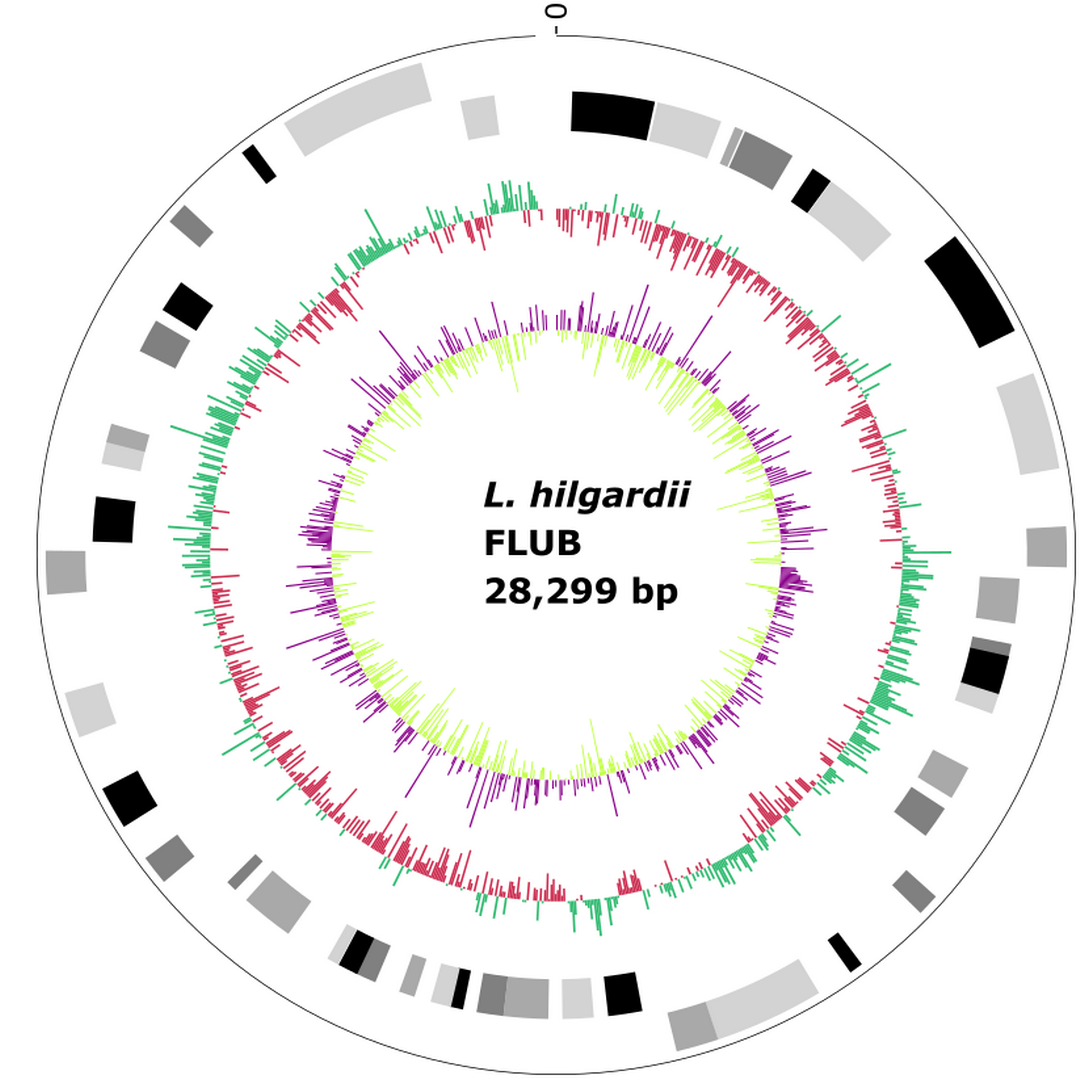

Supplement: Supplementary file 1 [file ijms-22-03780-s001.zip › Supplementary Materials/Supplementary Materials/CGView/CGView_plasmid3_L.hilgardii.tif]

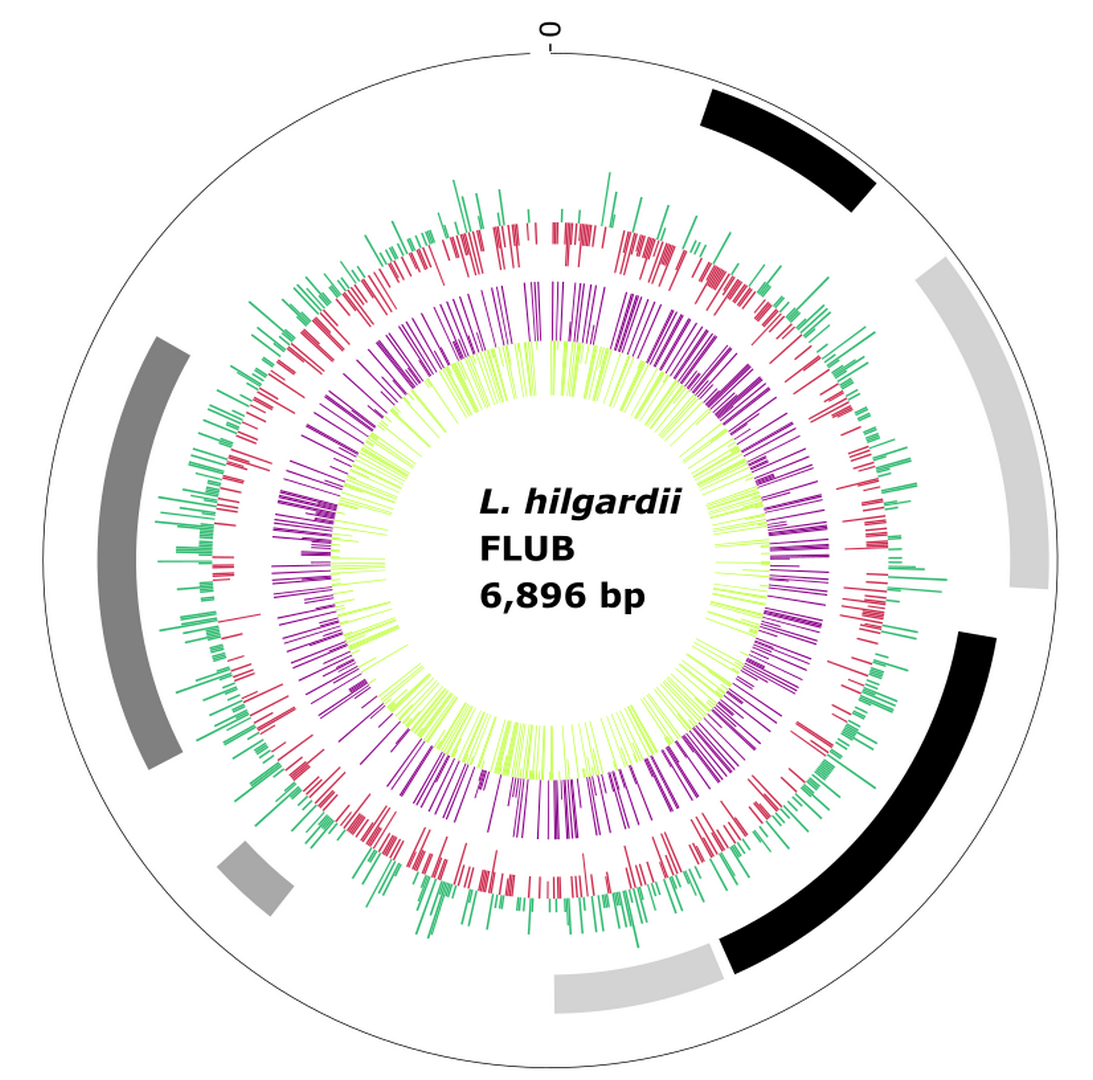

Supplement: Supplementary file 1 [file ijms-22-03780-s001.zip › Supplementary Materials/Supplementary Materials/CGView/CGView_plasmid4_L.hilgardii.tif]

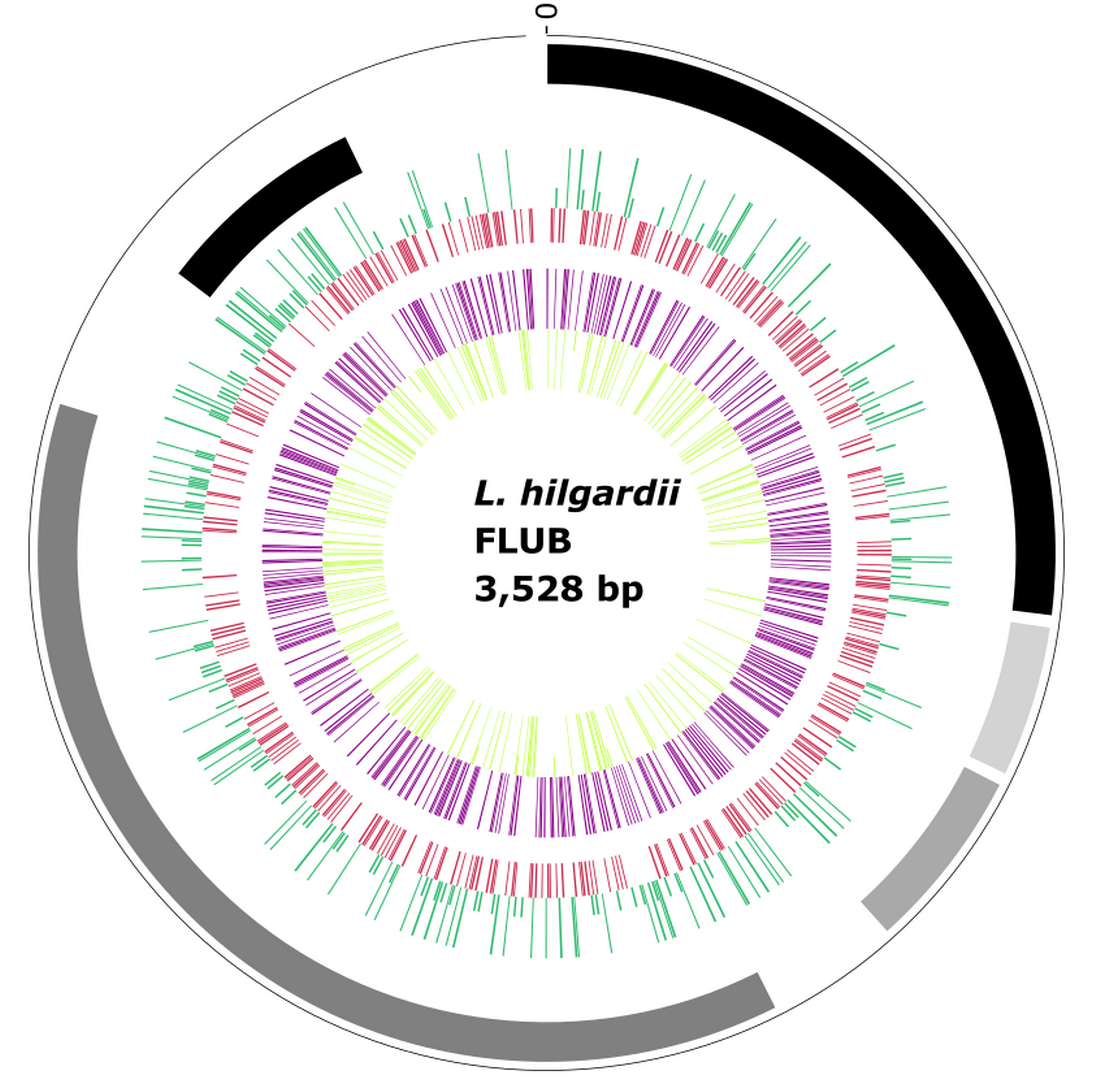

Supplement: Supplementary file 1 [file ijms-22-03780-s001.zip › Supplementary Materials/Supplementary Materials/CGView/CGView_plasmid5_L.hilgardii.tif]
